# Concerted Action of Targeted Nucleic Acid Therapeutics as Flexible, Precision and Personalized Cancer Treatment

**DOI:** 10.1002/advs.76840

**Published:** 2026-07-29

**Authors:** Jing Wu, Jiaxuan Chen, Abdechakour Elkihel, Wenjun Lan, Nicolas Fraunhoffer, Dandan Zhu, Baoping Lian, Christina Galanakou, Zhancun Bian, Xi Liu, Odile Gayet, Loic Moubri, Julie Aglietti Roques, Vikash Reebye, Nagy Habib, Xiaoxuan Liu, Nelson Dusetti, Juan Lucio Iovanna, Ling Peng

**Affiliations:** ^1^ Aix Marseille University, CNRS, Centre Interdisciplinaire de Nanoscience de Marseille (CINaM), UMR 7325 Equipe Labellisée Ligue Contre le Cancer Marseille France; ^2^ Aix Marseille University, CNRS, INSERM, Institut Paoli‐Calmettes CRCM Marseille France; ^3^ State Key Laboratory of Natural Medicines, Joint International Research Laboratory of Target Discovery and New Drug Innovation (Ministry of Education), Jiangsu Key Laboratory of Drug Discovery for Metabolic Diseases, Center of Advanced Pharmaceuticals and Biomaterials China Pharmaceutical University Nanjing China; ^4^ Center For Pharmacological and Botanical Studies, Faculty of Medicine, National Scientific and Technical Research Council University of Buenos Aires Buenos Aires Argentina; ^5^ MiNA Therapeutics Ltd, Translation & Innovation Hub London UK; ^6^ Department of Surgery and Cancer, Hammersmith Hospital Imperial College London London UK

**Keywords:** dendrimer nanovector, oncogene targeting, pancreatic cancer, small activating RNA, small interfering RNA, tumor suppressor activation

## Abstract

Nucleic acid therapeutics offer unique opportunities to develop personalized precision therapies by responding specifically and effectively at the gene level to evolving pathogens. However, many nucleic acid drugs employ a single‐target strategy, which is effective in monogenic disorders, yet can be ineffective in treating diseases characterized by complex and interacting molecular events. Nucleic acid combinations capable of simultaneously modulating multiple pathways and harnessing concerted effects constitute a promising approach to combatting complex, dynamically evolving and heterogeneous diseases. Here, we report a combination strategy utilizing small activating RNA to activate tumor suppressor genes alongside small interfering RNA to silence oncogenes for therapeutic potential and clinical relevance in cancer treatment using patient‐derived tumor models of pancreatic cancer. Transcriptomic and proteomic profiling of individual patient tumors enabled rational design of patient‐specific combinations, which exhibited superior anticancer efficacy through coordinated regulation of multiple genes. Importantly, diverse combinations were tailored to individual patient tumor models to elicit therapeutic responses, while certain specific combinations demonstrated therapeutic efficacy across different tumor models that shared similar genetic features, highlighting the flexibility and pan‐treatment potential. Our findings not only confirm the efficacy of the combination approach for cancer treatment, but also underscore its potential applicability to other complex malignancies.

## Graphical abstract

Combinations of small activating RNAs and small interfering RNAs were developed as personalized precision therapies that simultaneously activate tumor suppressors and silence oncogenes according to the molecular signatures of patient tumors. This combination strategy demonstrated superior anticancer efficacy, highlighting the promise of precision combinatorial nucleic acid therapeutics as flexible and personalized treatment.

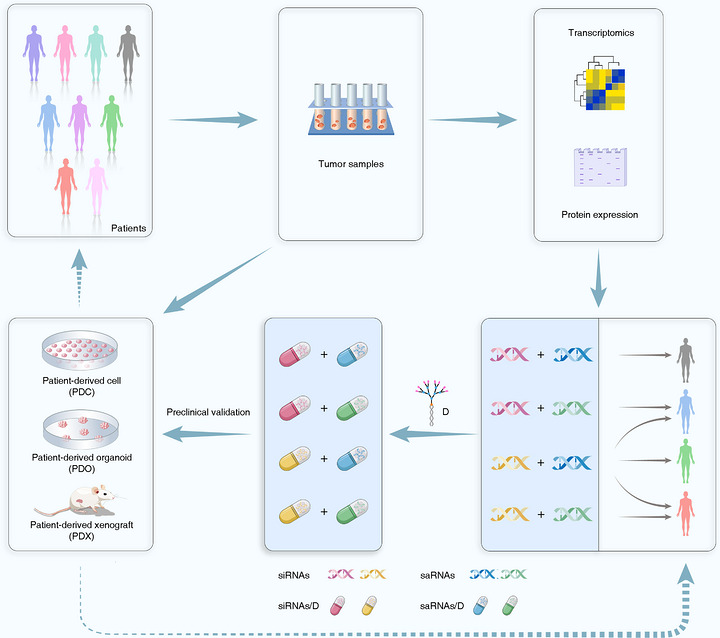

## 1 Introduction

The post‐genomic era has precipitated a paradigm shift toward precision medicine, with nucleic acid therapeutics presenting unique opportunities for developing precision and personalized medicine approaches [1, 2, 3]. Nucleic acid therapeutics respond rapidly and effectively at the gene level to evolving pathogens while addressing previously “undruggable” targets. They are able to modulate precisely gene expression in disease‐associated pathways, generating specific, effective and sustained therapeutic outcomes while minimizing off‐target effects. Their precise targeting capabilities, design flexibility, and rapid development timelines, offer a cutting‐edge technological platform for advancing precision and personalized medicine across diverse disease applications [1, 2, 3]. Particularly promising are small interfering RNA (siRNA) [4, 5] and small activating RNA (saRNA) [6, 7], which demonstrate distinct mechanisms of action and broad therapeutic potential across multiple disease categories. Mechanistically, siRNA cleaves the target mRNA through RNA interference (RNAi) to achieve potent and sequence‐specific gene silencing [4, 5], while saRNA, on the other hand, upregulates gene expression via binding to the promoter region of the target gene and recruiting the transcription machinery. This distinctive gene activation mechanism facilitates restoration of defective or inactivated critical signaling pathways and cellular functions, providing a novel therapeutic strategy for disease intervention [6, 7, 8]. To date, seven siRNAs have been approved by the U.S. Food and Drug Administration (FDA) [1], demonstrating the proven clinical efficacy of this next‐generation therapeutic modality for disease intervention. Additionally, two saRNAs have also entered clinical trials targeting multiple cancer types, demonstrating the translational potential of saRNA as an innovative strategy for cancer treatment [7].

Cancer represents a complex genetic disorder characterized by oncogene activation and tumor suppressor downregulation, with individual patients exhibiting distinct and evolving gene expression profiles from disease initiation and through development and progression. Given the potential of saRNAs to activate tumor suppressor genes and siRNAs to downregulate target oncogenes, these nucleic acid therapeutics constitute an optimal approach for precise and personalized cancer therapy. Specifically, the combination of saRNA and siRNA to simultaneously activate tumor suppressor genes while silencing oncogenes is anticipated to generate concerted therapeutic efficacy while reducing potential adverse effects and drug resistance risks, offering a novel and viable strategy for treating cancer, a particularly complex, heterogeneous and dynamically evolving disease.

To demonstrate the therapeutic potential of combined saRNA and siRNA approaches for personalized and precision cancer treatment, we conducted a proof‐of‐concept study using pancreatic cancer as the disease model. Pancreatic cancer, particularly, pancreatic ductal adenocarcinoma (PDAC), represents one of the most lethal malignancies, with a five‐year survival rate below 10% and no effective treatment options currently available [9]. PDAC is characterized as a highly complex disease with heterogeneity and phenotype alterations driven by multi‐gene interactions and dysregulated signaling pathways [10, 11]. Therefore, combination therapy approaches, such as saRNA combined with siRNA, which can simultaneously target multiple critical genes and pathogenic pathways, represent a promising strategy for personalized and precise treatment of PDAC.

**FIGURE 1:**
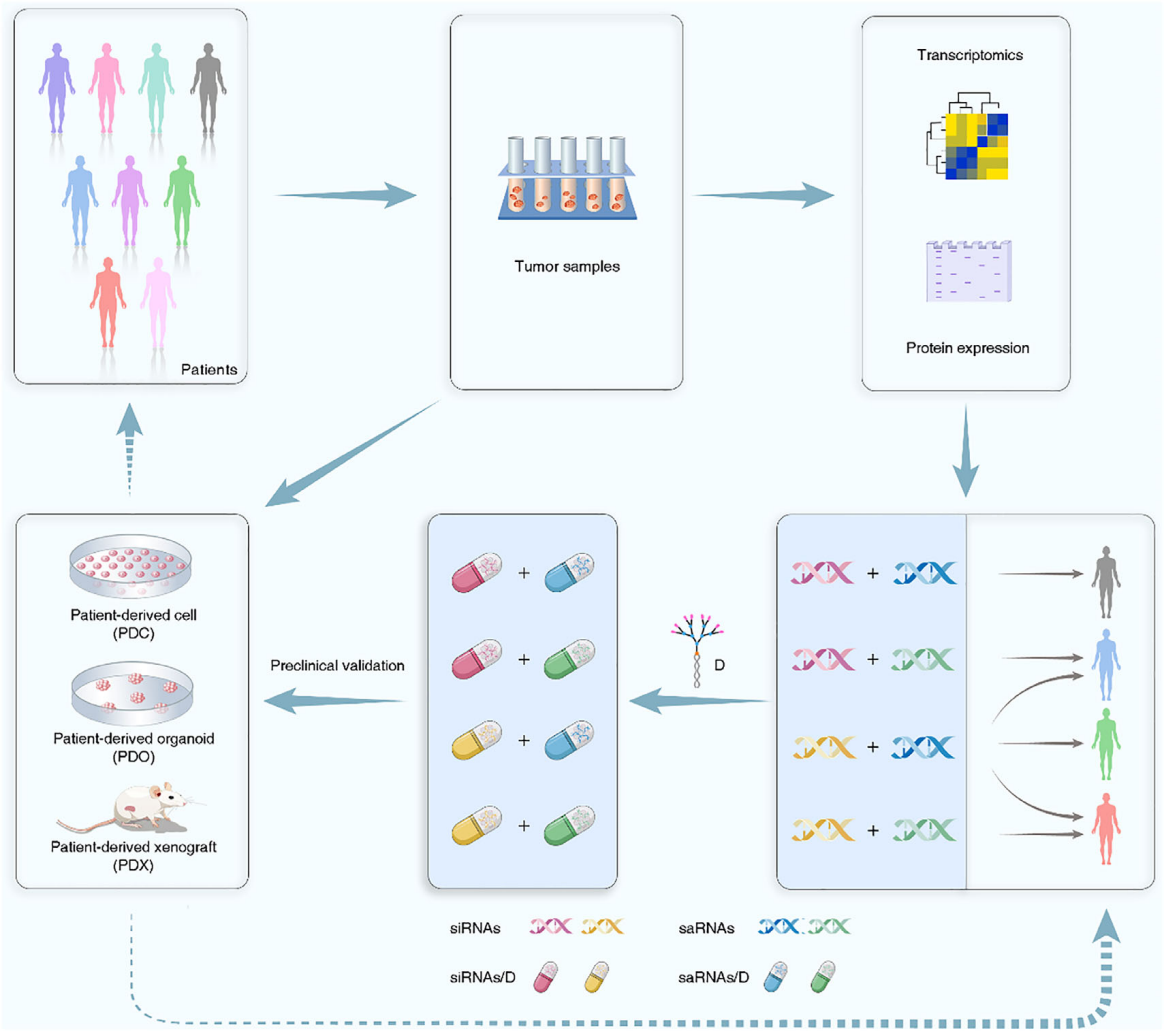
saRNA/siRNA combination as precision and personalized treatment strategy for pancreatic cancer. Schematic representation illustrating the rational design of saRNA/siRNA combinations based on transcriptomic analyses and protein expression profiling of patient tumor specimens to enable precision and personalized interventions, which are then formulated with the amphiphilic dendrimer vector and validated using patient‐derived primary pancreatic cancer cell culture (PDC), organoid (PDO) and tumor xenograft (PDX) models.

In this proof‐of‐concept investigation, we first selected key driver genes as targets in treating PDAC, then designed the saRNA/siRNA combinations based on transcriptomic analyses of 45 patient‐derived PDAC specimens and the related protein expression profile, to develop a precise and personalized PDAC treatment approach (Figure 1). To solve the problem of chemo‐ and enzymo‐lability as well as membrane impermeability inherent to nucleic acid therapeutics, we employed an amphiphilic dendrimer vector (D) for delivering saRNA/siRNA combinations, and evaluated the consequent gene regulation and therapeutic efficacy using patient‐derived primary pancreatic cancer cell culture (PDC), patient‐derived cancer organoid (PDO), and patient‐derived tumor xenograft (PDX) models. Our results demonstrate considerably enhanced anticancer activity exhibited by the saRNA/siRNA combinations compared to their monotherapy approaches. Importantly, multiple saRNA/siRNA combinations could be designed according to the transcriptomic and protein expression profiles of individual patient tumors to achieve effective therapeutic responses, demonstrating the flexibility of this precise treatment option. Also notably, specific combinations could generate therapeutic effects across multiple patient‐derived tumor models exhibiting similar target gene expression patterns, highlighting the pan‐therapeutic capacity. This study not only demonstrates the feasibility and efficacy of saRNA/siRNA combination approach for PDAC treatment, but also provides valuable insights applicable to other cancers and diseases characterized by complex gene regulatory networks.

## 2 Results

### 2.1 Selection of Key Driver Genes for saRNA/siRNA Combination Therapy in PDAC

PDAC pathogenesis is fundamentally driven by genetic alterations. Four well‐established driver genes: *KRAS* (Kirsten rat sarcoma viral oncogene homolog), *TP53* (tumor protein p53), *SMAD4* (SMAD family member 4), and *CDKN2A* (cyclin‐dependent kinase inhibitor 2A) have been intensively studied. Despite extensive exploration of therapeutic approaches targeting these genes, clinical success remains limited [10, 11, 12]. Currently, the FDA has approved only two targeted therapies for *KRAS* G12C mutations—sotorasib and adagrasib. Yet, neither of them has been approved for the treatment of pancreatic cancer, highlighting the critical need for therapeutic strategies against alternative molecular targets [13, 14].

Accumulating evidence implicates additional regulatory genes in PDAC tumorigenesis and malignant progression. For example, upregulated oncogenes in PDAC include: 1) *MYC*: a proto‐oncogene encoding the transcription factor MYC that regulates cell cycle progression, metabolism, and proliferation, and its overexpression is frequently observed in PDAC and drives rapid tumor growth [15, 16, 17]; 2) *AKT2*: a serine/threonine kinase gene and key component of the PI3K/AKT/mTOR signaling pathway, promotes cell survival, proliferation, and metastasis [18, 19]; 3) *BIRC5*: an anti‐apoptotic gene encoding Survivin, is often upregulated in PDAC and contributes to apoptosis evasion and tumor cell survival [20, 21]. Also, several tumor suppressor genes were found down‐regulated in PDAC, including: 1) *CDKN1A*: a tumor suppressor gene encoding the cyclin‐dependent kinase inhibitor p21, which suppresses tumor growth by arresting cell cycle progression [22, 23]; 2) *CEBPA* (CCAAT/enhancer‐binding protein alpha): a tumor suppressor gene encoding the transcription factor C/EBPα, which regulates cell proliferation and metastasis‐associated pathways [24, 25, 26]. More importantly, nucleic acid therapeutics targeting *MYC, BIRC5*, *CDKN1A* and *CEBPA* have clinically validated these four target genes, including: 1) DCR‐MYC: an siRNA targeting *MYC* [27], and its phase 1 trials being terminated by Dicerna Pharmaceuticals; 2) LY2181308: an antisense oligonucleotide targeting BIRC5 mRNA, entered into phase II clinical trials against various cancers after phase I study [28, 29]; 3) MTL‐CEBPA: an saRNA activating *CEBPA*, has progressed into phase II clinical trials in hepatocellular carcinoma [30], with additional clinical evaluation in combination with Pembrolizumab [31], Sorafenib [32], and Atezolizumab plus Bevacizumab [33]; 4) RAG‐01: an saRNA for activating *CDKN1A* expression, has advanced to a Phase II clinical trial for the treatment of non‐muscle‐invasive bladder cancer [34]. Based on these promising results, we wished to test the efficacy of the saRNA/siRNA combination approach in treating PDAC.

**FIGURE 2:**
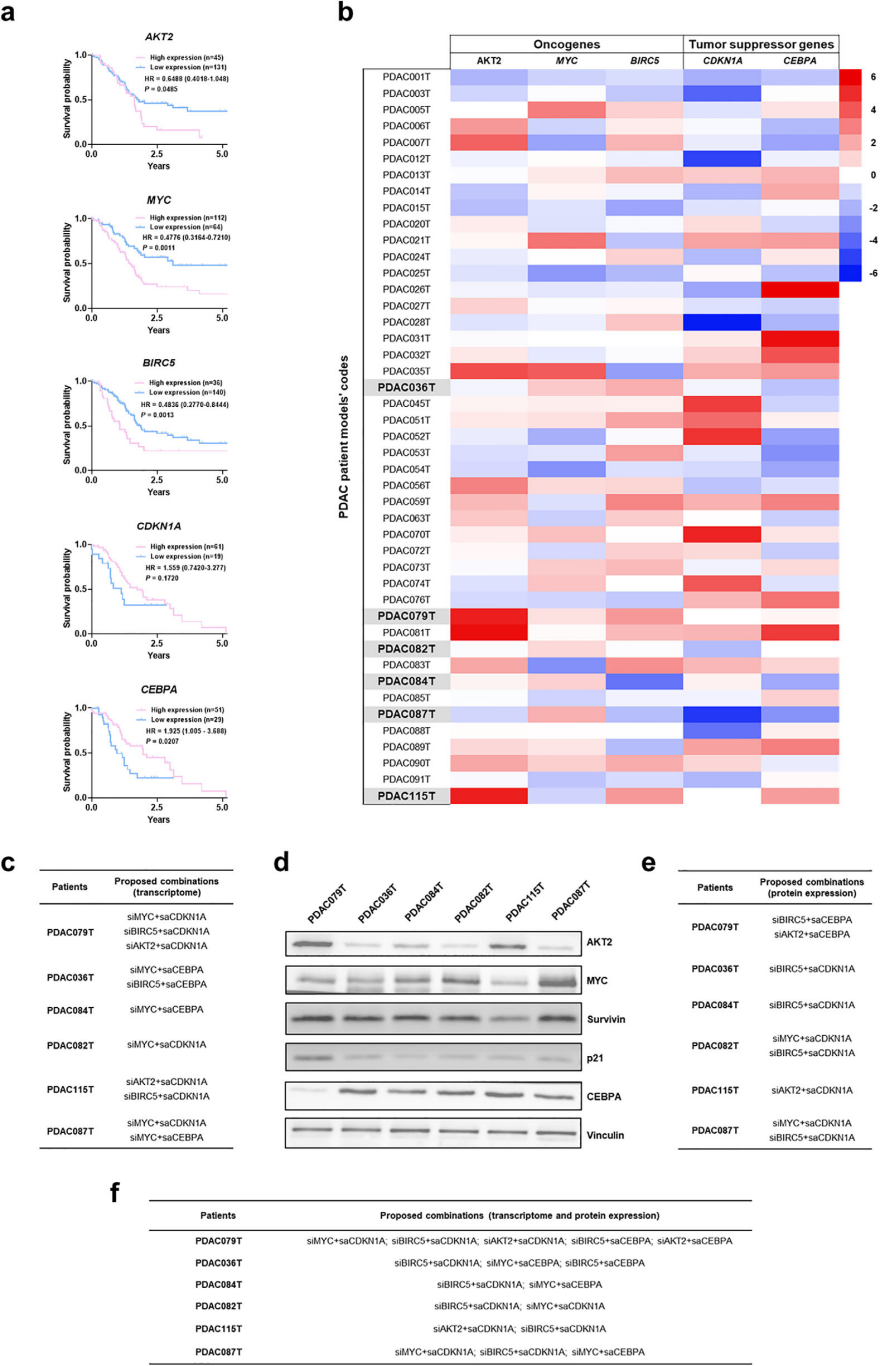
saRNA/siRNA combinations designed as precise and personalized therapy for PDAC. (a) Relationship between expression levels of *AKT2*, *MYC*, *BIRC5*, *CDKN1A*, and *CEBPA*, and overall survival in pancreatic cancer patients (Human Protein Atlas database) (HR:Hazard Ratio; *P*: Log‐rank *p*‐value). (b) Transcriptomic analysis of selected genes (*AKT2*, *MYC*, *BIRC5*, *CDKN1A*, and *CEBPA*) of 45 patient‐derived primary cells from tumor samples issued from the PaCaOmics clinical trial. (c) Proposed saRNA/siRNA combination treatments for the six selected tumor models (PDAC079T, PDAC115T, PDAC087T, PDAC082T, PDAC084T, and PDAC036T) based on transcriptomic analysis in b. (d) Protein expression profiles of AKT2, MYC and Survivin, p21 and CEBPA in the six selected patient tumor models. (e) Proposed saRNA/siRNA combination treatments based on protein expression levels in d. (f) Collective saRNA/siRNA combinations proposed as precision therapy for the six PDAC models by integrating their transcriptomic and protein expression profiles.

Prior to initiating this study, we analyzed the survival correlations of these five genes (*AKT2, MYC*, *BIRC5*, *CDKN1A*, and *CEBPA*) in PDAC using the Human Protein Atlas database (Figure 2a) [35]. Consistent with their biological functions, low expression of oncogenes *AKT2*, *MYC* and *BIRC5* was associated with favourable prognosis, while high expression of tumor suppressor genes *CDKN1A* and *CEBPA* significantly correlated with improved overall survival in pancreatic cancer patients (Figure 2a). The prognostic evidence provided strong rationale and support for targeting these genes in PDAC through our saRNA/siRNA combination approach.

### 2.2 Design of saRNA/siRNA Combinations for Precision and Personalized Therapy

To develop saRNA/siRNA combinations simultaneously targeting *AKT2*, *MYC*, *BIRC5*, *CDKN1A*, and *CEBPA* for precision and personalized therapeutic approaches in our proof‐of‐concept study, we first performed comprehensive transcriptomic analysis of these five genes across 45 patient‐derived primary pancreatic cancer cells issued from tumor samples from the PaCaOmics clinical trial NCT01692873 [36]. Notably, the expression levels of individual oncogenes and tumor suppressor genes exhibited considerable inter‐patient variability (Figure 2b), demonstrating the pronounced molecular heterogeneity characteristic of PDAC and underscoring the critical importance of personalized precision medicine.

Based on the transcriptomic analysis from each patient's tumor specimen, we designed patient‐specific saRNA/siRNA combinations aiming to precisely target deregulated genes simultaneously in individual patients. For example in the patient‐derived tumor model PDAC082T, mRNA expression analysis revealed elevated *MYC* expression and diminished *CDKN1A* expression. Accordingly, a targeted siMYC+saCDKN1A combination (siMYC: siRNA targeting *MYC*; saCDKN1A: saRNA targeting *CDKN1A*) was designed as an optimal precise therapeutic strategy for this specific patient tumor model. Interestingly, the PDAC087T model exhibited elevated *MYC* expression alongside reduced expression of both tumor suppressor genes *CDKN1A* and *CEBPA*. Therefore, two distinct therapeutic combinations—siMYC+saCDKN1A and siMYC+saCEBPA (saCEBPA: saRNA targeting *CEBPA*)—were proposed as potential personalized treatment strategies for PDAC087T model. For this proof‐of‐concept investigation, we selected six patient‐derived PDAC models: PDAC079T, PDAC115T, PDAC087T, PDAC082T, PDAC084T, and PDAC036T, all demonstrating elevated mRNA expression of at least one of the three oncogenes (*AKT2* /*MYC* /*BIRC5*) and diminished expression of at least one of the two tumor suppressor genes (*CDKN1A* /*CEBPA*); in addition, we had well‐established PDC, PDO and PDX models for these 6 PDAC models. It is also to note that individual mRNA expression profiles for each patient‐derived PDAC model allowed the design of at least one targeted saRNA/siRNA combination (Figure 2c).

To complete the transcriptomic findings and verify the proposed saRNA/siRNA combinations, we further assessed protein expression levels of AKT2, MYC, Survivin, p21 and CEBPA in these six selected PDAC models using western blot analysis (Figure 2d). Notably, while some protein expression levels correlated with transcriptomic data, others exhibited discordance. These discrepancies can be attributed to post‐translational regulatory mechanisms, including enhanced protein stability through post‐transcriptional modifications and differential translational efficiency [37, 38]. For instance, Survivin in the PDAC087T model and p21 in the PDAC079T model demonstrated high protein expression (Figure 2d) despite low mRNA expression (Figure 2b). Such findings underscore the complexity of gene expression regulation and emphasize the critical importance of integrating both transcriptomic and proteomic data in therapeutic strategy design.

We therefore integrated saRNA/siRNA combinations derived from transcriptomic analyses (Figure 2c) and protein expression profiles (Figure 2e), and further customized combinations for individual patients (Figure 2f). Interestingly, the combination of siBIRC5+saCDKN1A (siBIRC5: siRNA targeting *BIRC5*) was proposed in all six selected patient‐derived models, highlighting that specific therapeutic combinations may achieve broad efficacy across defined patient subsets sharing similar expression profiles of certain target genes.

### 2.3 Amphiphilic Dendrimer D for Safe and Effective Delivery of saRNA and siRNA

**FIGURE 3:**
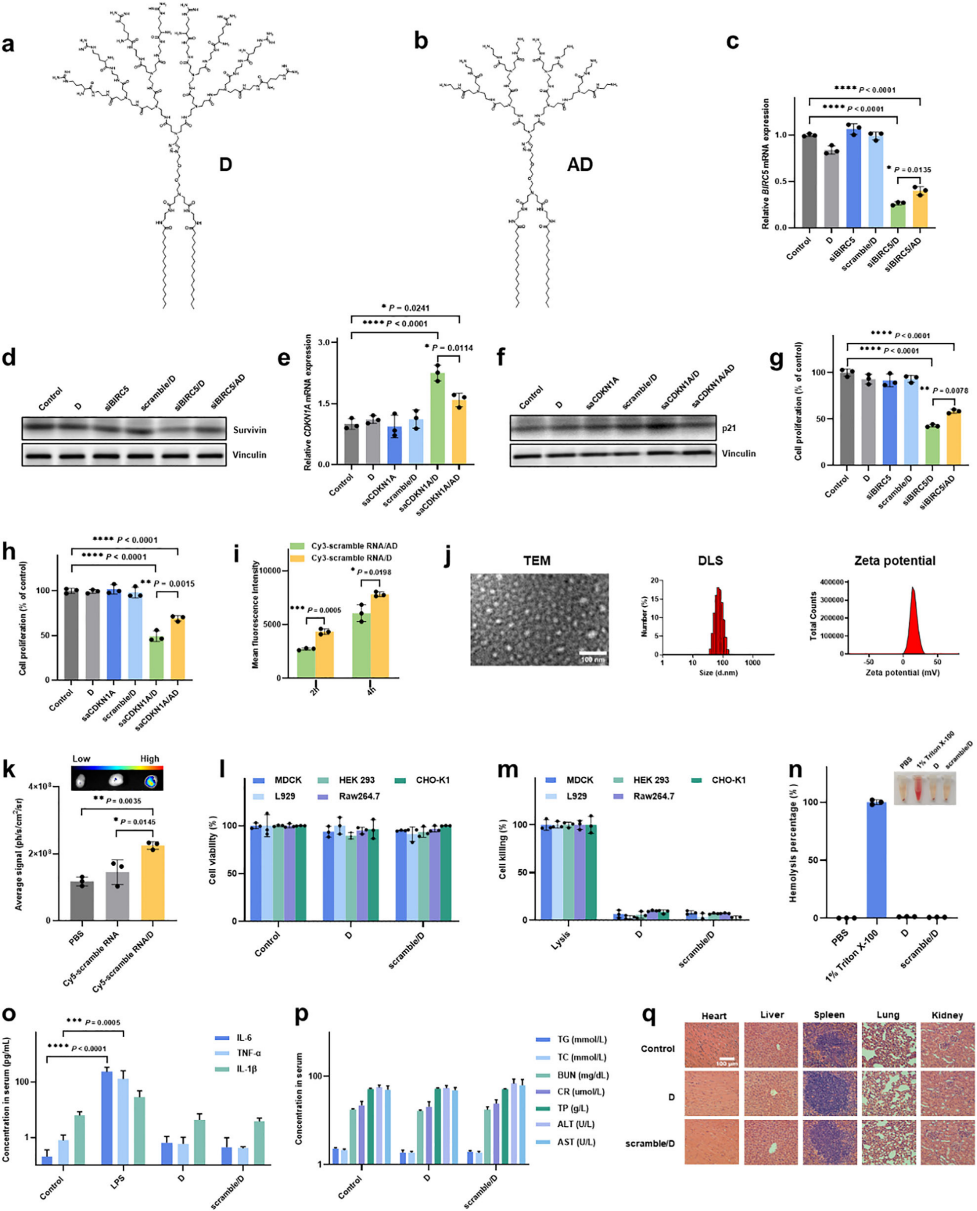
Dendrimer D enables effective RNA delivery with an excellent safety profile. (a) Chemical structure of the amphiphilic dendrimer D used in this study. (b) Chemical structure of the amphiphilic dendrimer AD previously reported [39]. (c) *BIRC5* mRNA expression (48 h post‐treatment) in PDAC087T cells treated with: non‐treatment (control), D alone, siBIRC5 alone, scramble RNA/D, siBIRC5/D and siBIRC5/AD. Data: mean ± standard deviation (SD) (*n* = 3). Significance by one‐way ANOVA/Tukey: *p < 0.05, ****p < 0.0001. (d) Survivin protein expression (72 h post‐treatment) in PDAC087T cells treated with the same conditions as in (c). (e) *CDKN1A* mRNA expression (48 h post‐treatment) in PDAC087T cells treated with: non‐treatment (control), D alone, saCDKN1A alone, scramble RNA/D, saCDKN1A/D and saCDKN1A/AD. Data: mean ± SD (*n* = 3). Significance by one‐way ANOVA/Tukey: *p < 0.05, ****p < 0.0001. (f) p21 protein expression (72 h post‐treatment) in PDAC087T cells treated with the same conditions as in (e). (g) Cell proliferation (MTT at 5 days post‐treatment) in PDAC087T treated with the same conditions as in (c). Data: mean ± SD (*n* = 3). Significance by one‐way ANOVA/Tukey: **p < 0.01, ****p < 0.0001. (h) Cell proliferation (MTT at 5 days post‐treatment) in PDAC087T treated with the same conditions as in (e). Data: mean ± SD (*n* = 3). Significance by one‐way ANOVA/Tukey: **p < 0.01, ****p < 0.0001. (i) Cellular uptake of Cy3‐labelled scramble RNA/D and Cy3‐labelled scramble RNA/AD complexes in PDAC087T cells (2 h / 4 h). Data: mean ± SD (*n* = 3). Significance by unpaired *t*‐test: *p < 0.05, ***p < 0.001. (j) Morphology, size and size distribution as well as zeta‐potential of the RNA/D complexes analyzed using TEM and DLS. (Scale bar for TEM image: 100 nm). (k) Tumor accumulation of Cy5‐labelled scramble RNA/D nanoparticles versus Cy5‐scramble RNA alone and PBS. (l) Metabolic cytotoxicity evaluation of dendrimer D and scramble RNA/D in MDCK, HEK293, CHO‐K1, L929, and Raw264.7 using MTT assay. (m) Membrane damage evaluation of D and scramble RNA/D on MDCK, HEK293, CHO‐K1, L929, and Raw264.7 cells using LDH release assay. (n) Hemolysis assay of D and scramble RNA/D on mouse red blood cells. (o) Serum inflammatory cytokines (IL‐1β, IL‐6, and TNF‐α) in mice treated with PBS (control), D alone, scramble RNA/D, with LPS positive control. Data: mean ± SD (*n* = 5). Significance by two‐way ANOVA/Dunnett: ***p < 0.001, ****p < 0.0001. (p) Blood biochemistry for liver/kidney function (ALT, AST, TG, TC, BUN, CR, and TP) in serum, mice treated with PBS (control), D or scramble RNA/D. (q) H&E staining of major organs from mice treated with PBS (control), D, scramble RNA/D. (Scale bar: 100 µm).

Based on the rationally designed saRNA/siRNA combinations as precision and personalized therapies, we wanted to evaluate the therapeutic efficacy of each saRNA/siRNA combination using the patient‐derived primary pancreatic cancer cell culture (PDC), patient‐derived cancer organoid (PDO), and patient‐derived tumor xenograft (PDX) models derived from tumor specimens in the PaCaOmics clinical trial [36]. However, the inherent challenges of nucleic acid molecules—namely easy degradation upon chemical/enzymatic action and poor permeability cross cell membrane—necessitate robust delivery vehicles. Here, we utilised an arginine‐bearing amphiphilic dendrimer vector D (Figure 3a; Figures S1 and S2) to deliver our saRNA/siRNA combinations. Dendrimer D is a modified variant of our previously reported dendrimer vector AD (Figure 3b) [39, 40], and exhibited a superior delivery efficacy for both siRNA and saRNA, leading to significantly more effective gene silencing (Figure 3c,d) and gene activation (Figure 3e,f) when compared to AD. By mimicking arginine‐rich cell penetrating peptides [41], D promoted higher cellular uptake than AD (Figure 3i), outperforming AD in delivering siRNA and saRNA with enhanced resulting biological consequence (Figure 3g,h).

Also importantly, the high aqueous solubility of dendrimer D enabled rapid and easy complex formation with RNA molecules by simple and brief vortexing in water at neutral pH, producing stable RNA/dendrimer complexes ready for use. This is a considerable advantage over the current lipid nanoparticles (LNPs)‐based delivery, which requires toxic organic solvents for microfluidic mixing at acid pH and subsequent removal of organic solvent and readjustment to neutral pH. The vortexing‐formed RNA/D complexes were uniform, spherical nanoparticles as demonstrated by using both transmission electron microscopy (TEM) analysis and dynamic light scattering (DLS) studies (Figure 3j). Importantly, these RNA/D complexes had weakly positive zeta‐potential (+14.8 mV) which is beneficial for minimizing nonspecific interactions with blood components, whereas the small size of these nanoparticles fell within the optimal range (typically 10–100 nm) for enhanced permeability and retention (EPR) effect [42, 43], favoring EPR‐based passive tumor targeting and accumulation within tumor tissues (Figure 3k). This passive tumor targeting capability is expected to ensure efficient intratumoural accumulation of the RNA/dendrimer complexes for subsequent therapeutic effects. Ex vivo fluorescence imaging of major organs confirmed the biodistribution profile (Figure S3a,b).

Further safety evaluations across multiple in vitro and in vivo models demonstrated excellent biocompatibility of the dendrimer vector D. MTT and LDH assays in different cells such as HEK293, MDCK, CHO‐K1, L929 and Raw264.7 cells showed no metabolic or membrane‐disruption associated toxicity from D or scramble RNA/D complexes (Figure 3l,m). Hemolysis assays in murine erythrocytes confirmed negligible red blood cell damage (Figure 3n). In healthy mice, treatment with D or scramble RNA/D showed cytokine profiles, blood biochemistry, and histology indistinguishable from PBS controls (Figure 3o–q), whereas the reference agent LPS induced marked inflammatory responses (Figure 3o). Collectively, these data highlight that amphiphilic dendrimer D provides a safe, user‐friendly, and highly efficient platform for delivering both saRNA and siRNA.

### 2.4 Concerted Gene Regulation and Enhanced Antiproliferative Activity in Primary PDC Models

**FIGURE 4:**
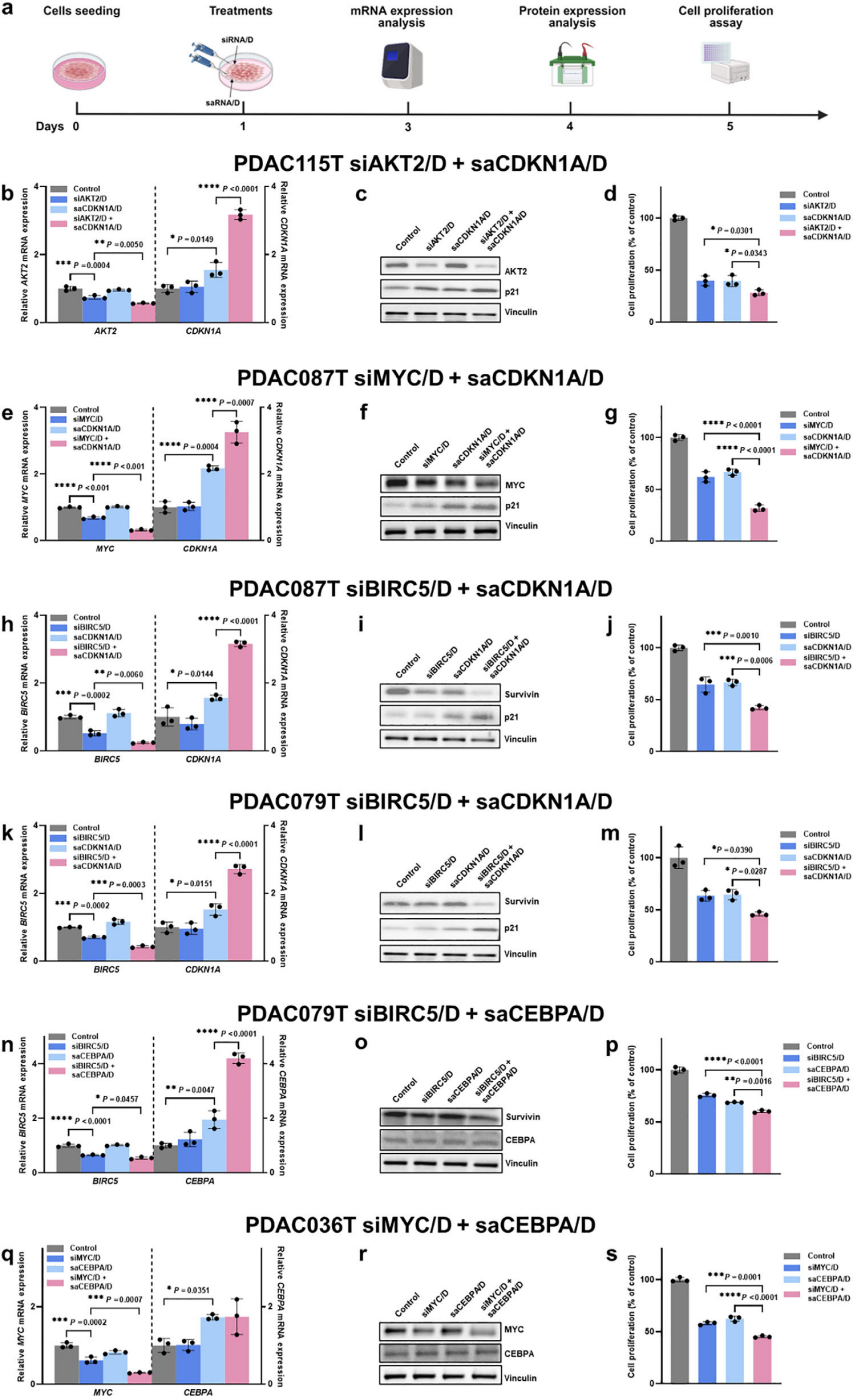
Evaluation of saRNA/siRNA combinations in PDC models for gene regulation and antiprolierative activity. (a) Schedule of cell assessment treated with saRNA/siRNA combination. (b–d) PDAC115T cells treated with non‐treatment (control), siAKT2/D, saCDKN1A/D, or combination: (b) *AKT2* and *CDKN1A* mRNA expression. (c) AKT2 and p21 protein expression. (d) Cell proliferation. (e–g) PDAC087T cells treated with non‐treatment (control), siMYC/D, saCDKN1A/D, or combination: (e) *MYC* and *CDKN1A* mRNA expression. (f) MYC and p21 protein expression. (g) Cell proliferation. (h–j) PDAC087T cells treated with non‐treatment (control), siBIRC5/D, saCDKN1A/D, or combination: (h) *BIRC5* and *CDKN1A* mRNA expression. (i) Survivin and p21 protein expression. (j) Cell proliferation. (k‐m) PDAC079T cells treated with non‐treatment (control), siBIRC5/D, saCDKN1A/D, or combination: (k) *BIRC5* and *CDKN1A* mRNA expression. (l) Survivin and p21 protein expression. (m) Cell proliferation. (n‐p) PDAC079T cells treated with non‐treatment (control), siBIRC5/D, saCEBPA/D, or combination: (n) *BIRC5* and *CEBPA* mRNA expression. (o) Survivin and CEBPA protein expression. (p) Cell proliferation. (q‐s) PDAC036T cells treated with non‐treatment (control), siMYC/D, saCEBPA/D, or combination: (q) *MYC* and *CEBPA* mRNA expression. (r) MYC and CEBPA protein expression. (s) Cell proliferation. After 48 h treatment, mRNA expression was quantified using qRT‐PCR. After 72 h treatment, protein expression was assessed using western blotting. At 5 days post‐treatment, cell proliferation was evaluated using MTT assay. For all treatments, siRNA or saRNA was used at a final concentration of 25 nM for single‐agent treatments, while combination treatments consisted of 25 nM siRNA and 25 nM saRNA. Data: mean ± SD (*n* = 3). Significance by one‐way ANOVA/Tukey: *p < 0.05, **p < 0.01, ***p < 0.001, ****p < 0.0001.

We next evaluated the therapeutic potential of the designed saRNA/siRNA combinations in patient‐derived primary pancreatic cancer cell culture (PDC) models using dendrimer D as the delivery vector [36], by assessing expression changes of targeted oncogenes and tumor suppressor genes at both mRNA and protein levels, alongside the resulting antiproliferative effects (Figure 4a).

Prior to validating the saRNA/siRNA combinations, we investigated the optimal strategy for constructing and delivering the combination treatment: 1) separately preparing saRNA/D and siRNA/D complexes followed by co‐administration; or 2) directly applying a single complex of (saRNA+siRNA)/D co‐loaded with both saRNA and siRNA. Although the first strategy enables flexible dosage adjustment, ratio optimization, and scheduling modifications for more precise and personalized therapy, the single complex approach offers advantages in preparation simplicity and formulation consistency. To determine the optimal strategy, we evaluated the delivery efficiency of siBIRC5+saCDKN1A in PDAC087T cells as a representative example. Our results revealed no significant differences between the two strategies in terms of silencing *BIRC5* and activating *CDKN1A* at both mRNA and protein levels, as well as the resulting antiproliferative activity (Figure S4a–c). Given the requirement for flexible dose modulation in clinical applications, we adopted the strategy of separately preparing saRNA/D and siRNA/D complexes for combination treatment in our subsequent studies, as this approach will enable independent adjustment of dosage and treatment schedule for each nucleic acid therapeutics, thereby optimally supporting personalized treatment concept and facilitating future clinical translation.

Employing the strategy of separately prepared saRNA/D and siRNA/D complexes, we evaluated all combinations proposed in Figure 2f across the six selected PDAC models. In the PDAC115T PDC model, the combinations of siAKT2/D+saCDKN1A/D (siAKT2: siRNA targeting *AKT2*) and siBIRC5+saCDKN1A both demonstrated superior efficacy compared to either monotherapy in gene regulation and consequent antiproliferative activity (Figure 4b–d and Figure S5a–c). For example, the combination of siAKT2/D+saCDKN1A/D reduced *AKT2* expression at both the mRNA and protein levels, with a silencing efficiency of approximately 45% vs siAKT2/D alone (25%) (Figure 4b,c). Also, this combination induced a ∼2.0‐fold increase in *CDKN1A* mRNA expression and led to a more pronounced activation at the protein level compared to saCDKN1A/D monotherapy (Figure 4b,c). This dual‐regulation strategy—simultaneously enhancing silencing of the oncogene *AKT2* while activating the tumor suppressor gene *CDKN1A*—resulted in approximately 70% inhibition of cell viability vs each monotherapy (Figure 4d). These findings clearly demonstrate that the siAKT2/D+saCDKN1A/D combination achieved superior efficiency in precise and personalized molecular correction in patient‐derived primary cancer cells of PDAC115T, thereby enabling enhanced therapeutic outcomes.

Similarly, we evaluated, for the PDAC087T model, three distinct combination regimens: siMYC/D+saCDKN1A/D (Figure 4e–g), siBIRC5/D+saCDKN1A/D (Figure 4h–j), and siMYC/D+saCEBPA/D (Figure S5d–f). All three combinations exhibited superior gene regulatory activity and enhanced antiproliferative effects compared to respective single‐agent treatments. Notably, the combination regimens siBIRC5/D+saCDKN1A/D and siMYC/D+saCDKN1A/D, although designed for PDAC087T, also demonstrated effective therapeutic efficacy in the PDAC079T model (Figure 4k‐m and Figure S5g–i). This cross‐model efficacy can be attributed to similar high expression levels of targeted oncogenes and comparable low expression levels of the targeted tumor suppressor gene between the two patient‐derived tumor models.

We further extended our validation to the remaining combinations listed in Figure 2f: in the PDAC079T model, we tested the combinations of siBIRC5/D+saCEBPA/D (Figure 4n–p), siAKT2/D+saCEBPA/D (Figure S5j–l) and siAKT2/D+saCDKN1A/D (Figure S5m–o); in PDAC036T, we assessed the combinations of siMYC/D+saCEBPA/D (Figure 4q–s), siBIRC5/D+saCDKN1A/D (Figure S5p–r), and siBIRC5/D+saCEBPA/D (Figure S5s–u); in PDAC084T, we evaluated siMYC/D+saCEBPA/D (Figure S5v–x) and siBIRC5/D+saCDKN1A/D (Figure S5y‐aa); and in PDAC082T, we assessed siBIRC5/D+saCDKN1A/D (Figure S5ab‐ad) and siMYC/D+saCDKN1A/D (Figure S5ae‐ag). All results demonstrated concerted gene regulation and superior therapeutic responses to saRNA/siRNA combinations compared to single‐agent treatments in these PDC models.

Collectively, all data obtained from PDC models consistently demonstrated that rationally designed saRNA/siRNA combinations, based on patient‐specific molecular profiles, achieved superior therapeutic efficacy compared to their corresponding monotherapies. Importantly, analysis on the combination index (*CI*) using HSA model revealed the effective synergistic effect for all the combinations studied, with *CI* being less than 1.0 (Table S2). Additional evaluation of the saRNA/D and siRNA/D combinations across a 7 × 7 concentration matrix (from 5 to 45 nM for both saRNA and siRNA) using the HSA model in SynergyFinder further confirmed their synergistic effect (Figure S6). All these findings underscore the critical importance of personalized therapeutic approaches and emphasize the necessity of tailoring treatment strategies according to individual patients' specific molecular characteristics for effective combination. Interestingly, the siBIRC5/D+saCDKN1A/D combination demonstrated therapeutic efficacy in all six selected patient‐derived models, highlighting the potential to achieve somehow pan‐treatment efficacy within defined patient subsets sharing similar gene expression profiles.

### 2.5 Enhanced Inhibition on Tumor Organoid Growth Through saRNA/siRNA Combination

**FIGURE 5:**
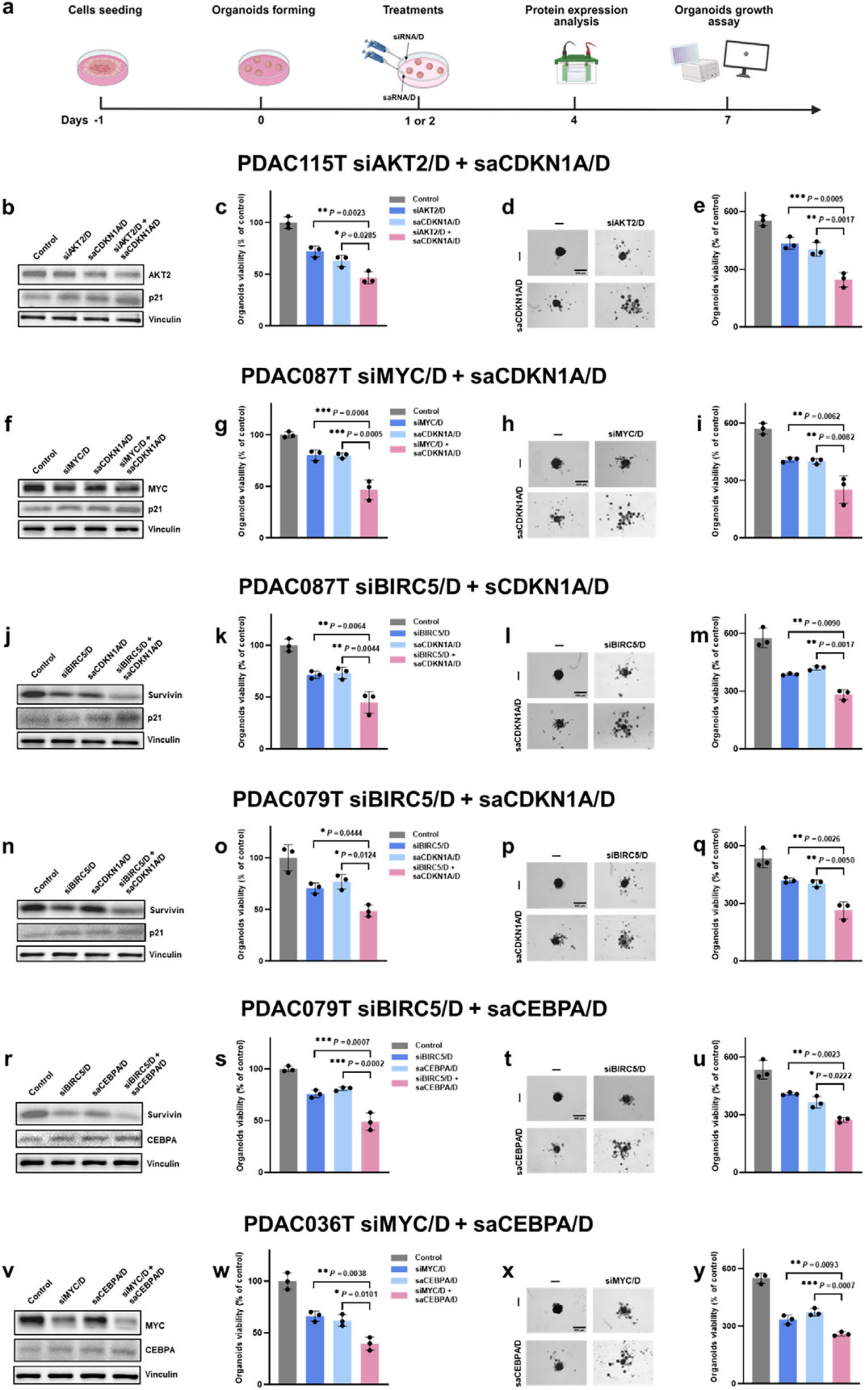
Evaluation of the saRNA/siRNA combinations in PDAC PDO models for gene regulation and organoid growth. (a) Timeline of organoid evaluation following saRNA/siRNA combination treatment. (b‐e) PDAC115T organoids treated with non‐treatment (control), siAKT2/D, saCDKN1A/D, or siAKT2/D+saCDKN1A/D combination: (b) AKT2 and p21 protein expression. (c) Organoid viability. (d) Organoid morphology. (e) Organoid diameter quantification from (d). (f‐i) PDAC087T organoids treated with non‐treatment (control), siMYC/D, saCDKN1A/D, or siMYC/D+saCDKN1A/D combination: (f) MYC and p21 protein expression. (g) Organoid viability. (h) Organoid morphology. (i) Organoid diameter quantification from (h). (j‐m) PDAC087T organoids treated with non‐treatment (control), siBIRC5/D, saCDKN1A/D, or siBIRC5/D+saCDKN1A/D combination: (j) Survivin and p21 protein expression. (k) Organoid viability. (l) Organoid morphology. (m) Organoid diameter quantification from (l). (n‐q) PDAC079T organoids treated with non‐treatment (control), siBIRC5/D, saCDKN1A/D, or siBIRC5/D+saCDKN1A/D combination: (n) Survivin and p21 protein expression. (o) Organoid viability. (p) Organoid morphology. (q) Organoid diameter quantification from (p). (r‐u) PDAC079T organoids treated with non‐treatment (control), siBIRC5/D, saCEBPA/D, or siBIRC5/D+ saCEBPA/D combination. (r) Survivin and CEBPA protein expression. (s) Organoid viability. (t) Organoid morphology. (u) Organoid diameter quantification from (t). (v‐y) PDAC036T organoids treated with non‐treatment (control), siMYC/D, saCEBPA/D, or siMYC/D+saCEBPA/D combination: (v) MYC and CEBPA protein expression. (w) Organoid viability. (x) Organoid morphology. (y) Organoid diameter quantification from (x). After 72 h treatment, protein expression was assessed using western blotting. At 7 days post‐treatment, organoid viability was evaluated using ATP‐based viability assay and organoid morphology assay at 7 days post‐treatment was examined by light microscopy. For all experiments, siRNA or saRNA was administered at 50 nM in single treatments, while combination treatments included both siRNA and saRNA at 50 nM each. Data: mean ± SD (*n* = 3). Significance by one‐way ANOVA /Tukey: *p < 0.05, **p < 0.01, ***p < 0.001. Scale bar: 650 µm.

Compared to traditional two‐dimensional cell culture system, three‐dimensional patient‐derived cancer organoid models are physiologically more relevant, preserve tumor complexity, and give more accurate predictions of drug efficacy [44, 45, 46, 47]. Utilizing the more relevant PDO models, we can systematically assess therapeutic effects to generate predictive data for translational research. We thus further evaluated the therapeutic effects of the saRNA/siRNA combinations in PDO models—established with the patient‐derived primary pancreatic cancer cells issued from patient‐derived tumor xenograft models from PaCaOmics clinical trials [36]—by assessing protein expression changes of target oncogenes and tumor suppressor genes, as well as treatment‐induced inhibition of organoid growth (Figure 5).

In the PDAC115T PDO model, both siAKT2/D+saCDKN1A/D and siBIRC5/D+saCDKN1A/D combinations exhibited enhanced therapeutic efficacy compared with their respective monotherapies (Figures 5b–e and Figure S7a–d). Focusing on siAKT2/D+saCDKN1A/D as an example, western blot analysis demonstrated that the combination treatment of siAKT2/D+saCDKN1A/D significantly enhanced AKT2 protein silencing and p21 protein activation compared to either monotherapy (Figure 5b). ATP‐based viability assays further revealed that the combination treatment led to a ∼50% reduction in organoid viability, markedly outperforming the single‐agent treatments of siAKT2/D (∼20% reduction) and saCDKN1A/D (∼30% reduction) (Figure 5c). This dual‐targeting strategy—simultaneously silencing the oncogene AKT2 and activating the tumor suppressor p21—effectively disrupted organoid viability and significantly suppressed their growth. Morphological analysis confirmed this effect: organoids in the combination group exhibited extensive structural disintegration, whereas those in the control group maintained intact, spheroid morphology (Figure 5d). Further quantitative analysis demonstrated that combination treatment led to an approximate 55% reduction in average organoid diameter, whereas monotherapy resulted in a reduction of only around 25% (Figure 5e). Collectively, these data demonstrate efficient molecular and functional modulation by siAKT2/D+saCDKN1A/D in PDAC115T PDOs.

We subsequently evaluated the performance of all the other combinations to confirm the therapeutic efficacy across different models.

1) In the PDAC087T PDO model, the combinations of siMYC/D+saCDKN1A/D (Figure 5f–i), siBIRC5/D+saCDKN1A/D (Figure 5j–m), and siMYC/D+saCEBPA/D (Figure S7e–h) exhibited enhanced targeted regulatory effects compared to monotherapies, with organoid growth inhibition rates of approximately 50%.

2) In the PDAC079T PDO model, the combinations of siBIRC5/D+saCDKN1A/D (Figure 5n–q), siBIRC5/D+saCEBPA/D (Figure 5r–u), siAKT2/D+saCEBPA/D (Figure S7i–l) and siAKT2/D+saCDKN1A/D (Figure S7m–p) more effectively silenced oncogene‐encoded proteins while concurrently activating tumor suppressors, compared to monotherapies. Evaluation of ATP levels and organoid morphology revealed that, compared to monotherapies (∼20% reduction in organoid growth), these combination treatments produced more pronounced inhibition of organoid growth (∼50%).

3) In the PDAC036T PDO model, the combinations such as siMYC/D+saCEBPA/D (Figure 5v–y), siBIRC5/D+saCEBPA/D (Figure S7q–t), and siBIRC5/D+saCDKN1A/D (Figure S7u–x) exhibited consistent regulation effect and robust treatment efficacy as those achieved in the corresponding cell model.

4) In the PDAC084T organoids, the combination of siMYC/D+saCEBPA/D resulted in approximately 25% greater inhibition of organoid growth compared to monotherapy (Figure S7y‐ab).

Importantly, the obtained results demonstrate not only synergistic effect of the combination treatments (Table S3) but also strong consistency between PDOs and PDCs, highlighting the reliability of the molecular‐guided therapeutic strategies and the excellent cross‐model efficacy. It is also to mention that some combinations effective in PDCs showed inconsistent efficacy in PDOs (e.g., siMYC/D+saCDKN1A/D in PDAC079T; siBIRC5/D+saCDKN1A/D in PDAC084T; both siMYC/D+saCDKN1A/D and siBIRC5/D+saCDKN1A/D combinations in PDAC082T). These discrepancies likely stem from differential gene expression regulation between cell culture systems as reported for key signalling pathways and drug response genes. The greater physiological relevance of PDO gene expression profiles underscores their importance for clinical outcome prediction [46, 47]. Notably, no combinations effective in PDAC082T PDCs worked in corresponding PDOs, potentially reflecting unique microenvironmental influences [48].

As observed in PDC models, individual PDO models responded to multiple saRNA/siRNA combinations, highlighting multiple gene‐driven oncogenic mechanisms within tumors. This demonstrates the advantage of multi‐targeted therapeutic strategies, offering flexible treatment options. Similarly again, pan‐effective combinations like siBIRC5/D+saCDKN1A/D showed efficacy across multiple PDO models demonstrating that RNA‐based therapies can provide precision intervention for patient subgroups sharing similar pathway dysregulation. These findings significantly strengthen the translational rationale for saRNA/siRNA combination therapy.

### 2.6 Improved Therapeutic Efficacy of saRNA/siRNA Combinations in PDX Models

**FIGURE 6:**
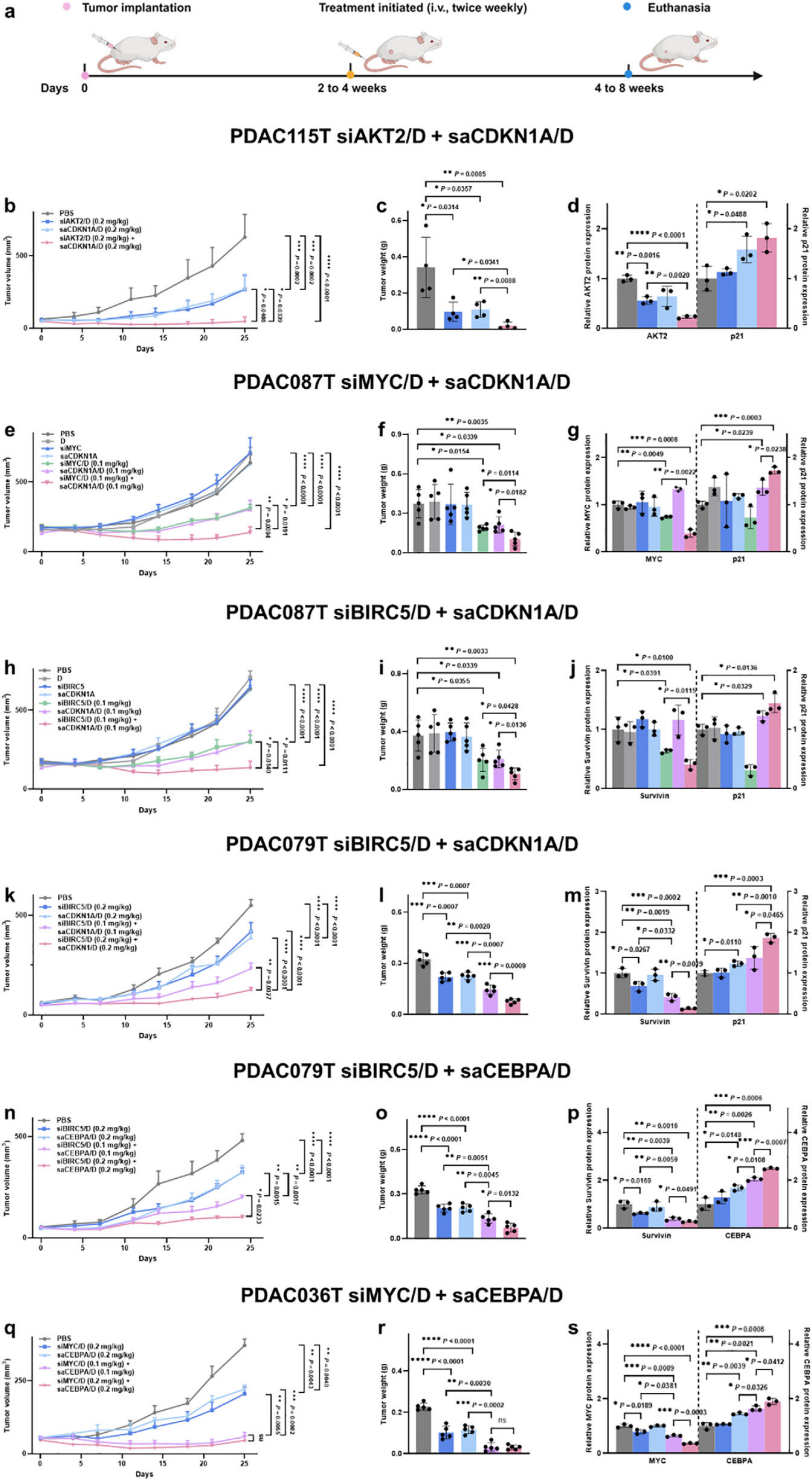
Evaluation of saRNA/siRNA combinations in PDX models for tumor growth inhibition and related gene regulation. (a) PDX model establishment and treatment schedule. (b–d) PDAC115T PDX mice treated with PBS (control), siAKT2/D (0.2 mg kg^−1^ siAKT2), saCDKN1A/D (0.2 mg kg^−1^ saCDKN1A), or combination (0.2 mg kg^−1^ each RNA) (*n* = 4): (b) Tumor growth. (c) Final tumor weight. (d) Protein expression of AKT2 and p21. (e–g) PDAC087T PDX mice treated with PBS (control), siMYC/D (0.1 mg kg^−1^ siMYC), saCDKN1A/D (0.1 mg kg^−1^ saCDKN1A), or combination (0.1 mg kg^−1^ each RNA) (*n* = 5): (e) Tumor growth. (f) Final tumor weight. (g) Protein expression of MYC and p21. (h–j) PDAC087T PDX mice treated with PBS (control), siBIRC5/D (0.1 mg kg^−1^ siBIRC5), saCDKN1A/D (0.1 mg kg^−1^ saCDKN1A), or combination (0.1 mg kg^−1^ each RNA) (*n* = 5): (h) Tumor growth. (i) Final tumor weight. (j) Protein expression of Survivin and p21. (k–m) PDAC079T PDX mice treated with PBS (control), siBIRC5/D (0.2 mg kg^−1^ siBIRC5), saCDKN1A/D (0.2 mg kg^−1^ saCDKN1A), or combination (0.1 or 0.2 mg kg^−1^ each RNA) (*n* = 5): (k) Tumor growth. (l) Final tumor weight. (m) Protein expression of Survivin and p21. (n–p) PDAC079T PDX mice treated with PBS (control), siBIRC5/D (0.2 mg kg^−1^ siBIRC5), saCEBPA/D (0.2 mg kg^−1^ saCEBPA), or combination (0.1 or 0.2 mg kg^−1^ each RNA) (*n* = 5): (n) Tumor growth. (o) Final tumor weight. (p) Protein expression of Survivin and CEBPA. (q–s) PDAC036T PDX mice treated with PBS (control), siMYC/D (0.2 mg kg^−1^ siMYC), saCEBPA/D (0.2 mg kg^−1^ saCEBPA), or combination (0.1 or 0.2 mg kg^−1^ each RNA) (*n* = 5): (q) Tumor growth. (r) Final tumor weight. (s) Protein expression of MYC and CEBPA. Data: Tumor growth panels (b, e, h, k, n, q): two‐way ANOVA/Tukey, mean ± SEM. Final weight panels (c, f, i, l, o, r): two‐tailed *t*‐test, mean ± SD. Other panels (d, g, j, m, p, s): two‐tailed *t*‐test, mean ± SD. Significance: *p < 0.05, **p < 0.01, ***p < 0.001, ****p < 0.0001.

Motivated by the demonstrated efficacy in the PDC and PDO models alongside the validated biocompatibility and tumor‐targeting of the dendrimer vector D, we further evaluated the antitumor efficacy of the saRNA/siRNA combinations in patient‐derived tumor xenograft (PDX) models. Specifically, we generated PDX models using Swiss nude or NOD/SCID mice bearing PDAC implants via subcutaneously injected patient‐derived primary pancreatic cancer cells or tumor mass [36, 49]. These PDX mice received twice‐weekly intravenous co‐administration of separately prepared saRNA/D and siRNA/D complexes (Figure 6a). The dose selection of siRNA or saRNA was primarily based on the results of the in vitro experiments alongside the preliminary data from animal experiments to ensure that single‐agent treatments were effective, while avoiding excessively high doses that could lead to near‐maximal or saturated responses and thereby obscure potential combinational effects.

In the PDAC115T PDX model, monotherapies with siAKT2/D or saCDKN1A/D at 0.20 mg kg^−1^ RNA dose inhibited tumor growth by more than 50% and reduced tumor weight by over 70% (Figure 6b,c), highlighting both the effectiveness of the selected gene targets and the efficiency of the dendrimer D delivery system. Importantly, combination therapy achieved over 90% inhibition of tumor growth (Figure 6b) and tumor weight reduction (Figure 6c), demonstrating a markedly enhanced effect. Protein expression analysis confirmed significantly enhanced downregulation of AKT2 and upregulation of p21 with the combination compared to single‐agent treatments (Figure 6d). Immunohistochemical analysis revealed a substantial reduction in Ki67‐positive cells, increased cleaved caspase‐3 expression, and enhanced apoptosis as indicated by TUNEL staining (Figure S8a), all indicating strong inhibition of tumor proliferation and promotion of apoptosis.

Similarly, in the PDAC087T PDX model, the siMYC/D+saCDKN1A/D combination produced more pronounced effects than either monotherapy, with tumor growth inhibition or tumor weight reduction exceeding 75% compared to approximately 50% with single agents at 0.10 mg kg^−1^ RNA dose (Figure 6e,f). Further molecular analyses showed effective downregulation of MYC and upregulation of p21 (Figure 6g), while immunohistochemistry and TUNEL staining confirmed superior inhibition of tumor proliferation and enhanced apoptosis (Figure S8b).

To further demonstrate therapeutic flexibility, we assessed the siBIRC5/D+saCDKN1A/D combination in the same PDAC087T PDX model. Single‐agent treatments with siBIRC5/D or saCDKN1A/D at 0.10 mg kg^−1^ RNA dose significantly inhibited tumor growth by ∼57% and reduced tumor weight by ∼50% (Figure 6h,i), while the combination treatment provided even greater efficacy. Protein expression analysis showed downregulation of Survivin and upregulation of p21 (Figure 6j), and immunohistochemical and TUNEL staining confirmed enhanced tumor suppression and apoptosis (Figure S8c).

Based on the in vitro results in the PDC and PDO models, the combination of siBIRC5/D+saCDKN1A/D also demonstrated strong anti‐proliferative and anti‐organoid growth effects in PDAC079T. We therefore also evaluated its dose‐dependent combination effects in the PDAC079T PDX model to validate its applicability across different patient tumor models. Here, single‐agent treatments at 0.20 mg kg^−1^ RNA dose inhibited tumor growth by only 25% and reduced tumor weight by around 30% (Figure 6k,l), whereas the combination (0.10 mg kg^−1^ each RNA) achieved 58% tumor growth inhibition and over 56% tumor weight reduction, outperforming both single‐agent treatments using either siRNA or saRNA. Doubling the dosage to 0.20 mg kg^−1^ of each RNA allowed even greater inhibition, up to 77%. Western blot analysis confirmed dose‐dependent therapeutic effects through downregulation of Survivin and upregulation of p21 (Figure 6m), which was further supported by reduced Ki‐67 and increased cleaved caspase‐3 expression detected by immunohistochemistry, along with enhanced TUNEL staining (Figure S8d).

We further evaluated the dose‐dependent combination effect of siBIRC5/D+saCEBPA/D and siMYC/D+saCEBPA/D in the PDX models of PDAC079T and PDAC036T, respectively. Both combinations significantly inhibited tumor growth and reduced tumor weight compared to single‐agent treatments (Figure 6n–o, and q,r). Immunohistochemistry and TUNEL staining confirmed improved inhibition of tumor proliferation and enhanced apoptosis (Figure S8e,f), while protein expression studies showed efficient downregulation of Survivin or MYC and upregulation of CEBPA (Figure 6p,s).

Throughout all i*n vivo* tests, mice maintained stable body weight and showed no signs of pathology or tissue damage (Figure S9a–l), confirming the safety of the dendrimer delivery system and the small RNA molecules used. In addition, all combination treatments exhibited synergistic effect as revealed by the values of the combination index (CI < 1.0) calculated using both HSA and Bliss models (Table S4). Collectively, our study systematically validated the superior and concerted anticancer effects of saRNA/siRNA combination therapy over single‐agent treatment using various PDX models. Notably, we assessed five distinct saRNA/siRNA combinations across four PDX models, all of which demonstrated therapeutic efficacy consistent with prior findings in PDC and PDO models. This high degree of cross‐model consistency not only confirms the translatability from in vitro to in vivo studies, but also considerably enhances the credibility of these therapeutic strategies for clinical translation. These findings provide robust preclinical evidence supporting the benefits of saRNA/siRNA combination treatment and underscore its substantial translational potential as a novel, flexible, precise, and personalized cancer therapy.

## 3 Conclusions

In this study, we present for the first time a saRNA/siRNA combination strategy that simultaneously silences and activates disease‐associated genes, offering a flexible, precise, and personalized therapeutic approach. Utilizing patient‐derived primary cancer cell cultures, organoids, and tumor xenograft models of pancreatic cancer—the most lethal malignancy—we demonstrated an enhanced and synergistic anticancer effect from the dual action of oncogene silencing and tumor suppressor gene activation achieved using the combination regimens compared to single‐agent treatments. Specifically, combination therapy more effectively inhibited tumor cell proliferation (measured by metabolic activity assays), organoid growth (assessed through metabolic and morphological analyses), and tumor progression (evaluated by tumor volume, tumor weight, Ki67, cleaved caspase‐3, and TUNEL assays) than monotherapies.

Critically, the design and validation of different saRNA/siRNA combinations for eliciting therapeutic responses within the same patient‐derived tumor model highlights the presence of multiple gene‐driven mechanisms in individual patients and underscores the promise of flexible, adaptive, multi‐targeted precision and personalized therapies. Additionally, certain specific combinations demonstrated consistent therapeutic efficacy across different patient derived tumor models, emphasizing the potential to tailor RNA‐based therapies to patient subsets characterized by similar signaling pathway dysregulations. All these findings provide promising perspective for guiding personalized precision therapy for patients from whom the tumor models were derived.

Specifically, in this study, we selected oncogenes (*AKT2, MYC*, and *BIRC5*) and tumor suppressor genes (*CDKN1A* and *CEBPA*) as targets to evaluate the feasibility of the concept of the saRNA/siRNA combination approach alongside the therapeutic impact against PDAC. These selected targets represent key oncogenic drivers and tumor suppressor pathways that play critical roles in the initiation and progression of PDAC. As PDAC is a highly complex and heterogeneous disease involving multi‐gene dysregulation and multiple pathogenic pathways, these selected targets may not fully encompass the molecular complexity of PDAC. Further or alternative targets alongside pathway‐level investigation could and will be explored in future studies to further expand the translational applicability of this approach.

It is also to note that we used the amphiphilic dendrimer as vector for saRNA/siRNA delivery in this study. Distinctly different from the clinically relevant delivery systems such as lipid nanoparticles (LNPs), the dendrimer nanovectors offer multiple unique advantages such as one‐component, precise structure, water solubility, complexing nucleic acids via simple vortexing at neutral pH, as well as safe and effective delivery [50, 51]. As the primary objective of this proof‐of‐concept study was to validate the concept of the saRNA/siRNA combination strategy, the use of amphiphilic dendrimer as the delivery vector offers a safe, effective and user‐friendly platform for evaluation of the saRNA/siRNA combinations in different PDAC models including PDC, PDO and PDX. In future translational context, it will be interesting to directly compare our platform with LNPs, the current benchmark for RNA delivery.

More importantly, our siRNA/saRNA combination approach clearly differs from existing RNA‐based combination strategies. While a variety of RNA combinations have been explored (e.g., siRNA+siRNA, miRNA‐based combinations, or mRNA‐based approaches), most of these strategies operate in the same direction (either activation or silencing). In contrast, our approach integrates gene activation (saRNA) and gene silencing (siRNA), enabling simultaneous upregulation of tumor suppressor genes and downregulation of oncogenes. This dual‐directional regulation allows correction of both upregulated and downregulated disease‐associated pathways, which cannot be achieved by conventional RNA combinations that rely on a single mode of action. Our siRNA/saRNA combination approach therefore offers a much more flexible and versatile application scenarios in therapeutic intervention based on nucleic acid therapeutics. This study has therefore not only introduced and validated a novel and effective siRNA/saRNA therapeutic strategy for pancreatic cancer but also highlights its potential for targeting multiple genes as a versatile precision medicine strategy for other malignancies and diseases with complex pathological regulatory landscapes.

## Author Contributions

**L.P**. conceived and coordinated the project, **J.C**. and **A.E**. performed dendrimer synthesis and characterization, **C.G**. performed TEM experiments, **J.W**., **D.Z**., **B.L**., **C.G**., **W.L**., **Z.B**., and **X.L**. performed the biological evaluation, **N.F**., **O.G**., **L.M**., **J.A.R**., **N.D**. and **J.L.I**., provided gene transcriptomic information and cell/organoids/PDX models from PaCaOmics clinical trials, **V.R**. and **N.H**. provided information of saCEBPA, **J.W**., **J.C**., **W.L**., **D.Z**., and **L.P**. analyzed the data, **J.W**. and **L.P**. wrote the manuscript with comments from all co‐authors.

## Conflicts of Interest

NH co‐founded MiNA Therapeutics (https://minatx.com/) in 2008 and was involved in the company until 2023. VR has equity in MiNA Therapeutics Ltd. The other authors declare no competing interests

## Supporting information




**Supporting File**: advs76840‐sup‐0001‐SuppMat.pdf.

## Data Availability

The data that support the findings of this study are available on request from the corresponding author. The data are not publicly available due to privacy or ethical restrictions.
