# Supplementary material for: Concerted Action of Targeted Nucleic Acid Therapeutics as Flexible, Precision and Personalized Cancer Treatment
Source: Adv Sci (Weinh). 2026 Jul 29:e76840. Online ahead of print. doi: 10.1002/advs.76840 (PMC13418047; doi:10.1002/advs.76840)
Supplement: Supplementary file 1 — Supporting File: advs76840‐sup‐0001‐SuppMat.pdf. [file ADVS-9999-e76840-s001.pdf]

Supplementary Materials

Table of Content

Fig. S1 3
Fig. S2 4
Fig. S3 5
Fig. S4 6
Fig. S5 7
Fig. S6 9
Fig. S7 11
Fig. S8 13
Fig. S9 14
Table S1 16
Table S2 17
Table S3 18
Table S4 19
Materials and methods 20
Synthesis and characterization of the dendrimer D 20
Transmission electron microscopy (TEM) 20
Dynamic light scattering (DLS) analysis 20
RNA/dendrimer complex formation 21
Cells 21
Preclinical PDAC models 21
Primary patient-derived pancreatic cancer cells 21
Patient-derived pancreatic cancer organoids 22
Cellular uptake 22
MTT assay for dendrimer D cytotoxicity 22
Lactate dehydrogenase (LDH) assay 22
Hemolysis assay 22
In vitro transfection on patient-derived cells 23
Synergy assessment of combinations with varying concentrations in matrix in PDCs 23
In vitro transfection on patient-derived organoids 23
MTT assay for antiproliferation of RNA/dendrimer complexes 24
CellTiter-Glo® 3D Cell Viability assay 24
Assessment of organoid morphology 24
Western blot analysis 24
Quantitative real-time (qRT)-PCR analysis 24
Animals 25
Patient-derived xenograft models 25
Biodistribution of the RNA/D complex in PDX model 25
Antitumor activity evaluation of combination treatments in PDX models 25
Inflammatory Cytokine Assay 26
Biochemical Factor Assay 26

Hematoxylin and eosin Staining........26
TUNEL Assay........26
Immunohistochemistry........26
Combination index analysis based on HSA and Bliss models........27
Statistical tests........27
References........27

Fig. S1. Synthesis of the amphiphilic dendrimer D.

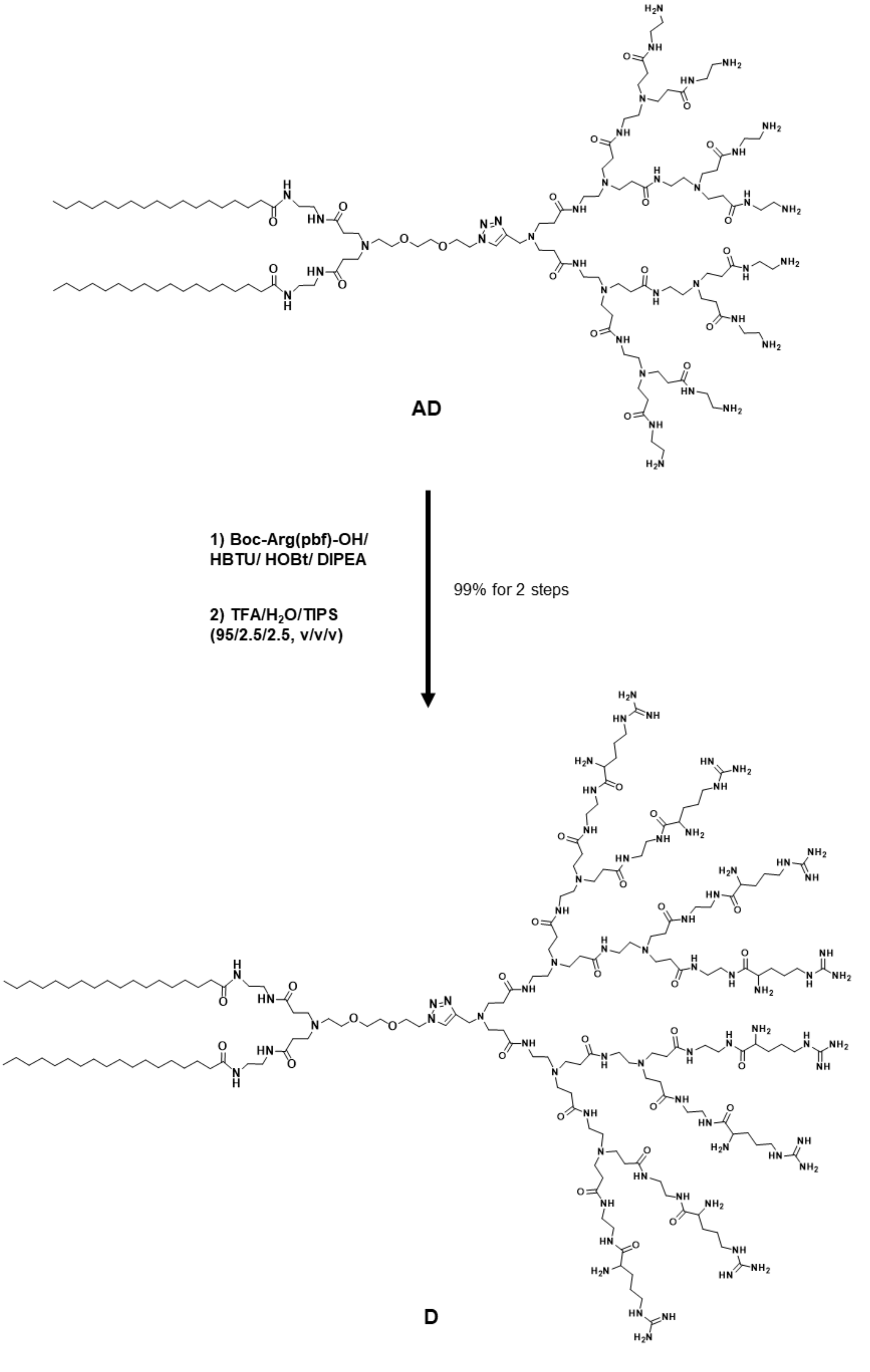

Fig. S2. Structural characterization of D. (a) ^{1}H-NMR, (b) ^{13}C-NMR and (c) HRMS.

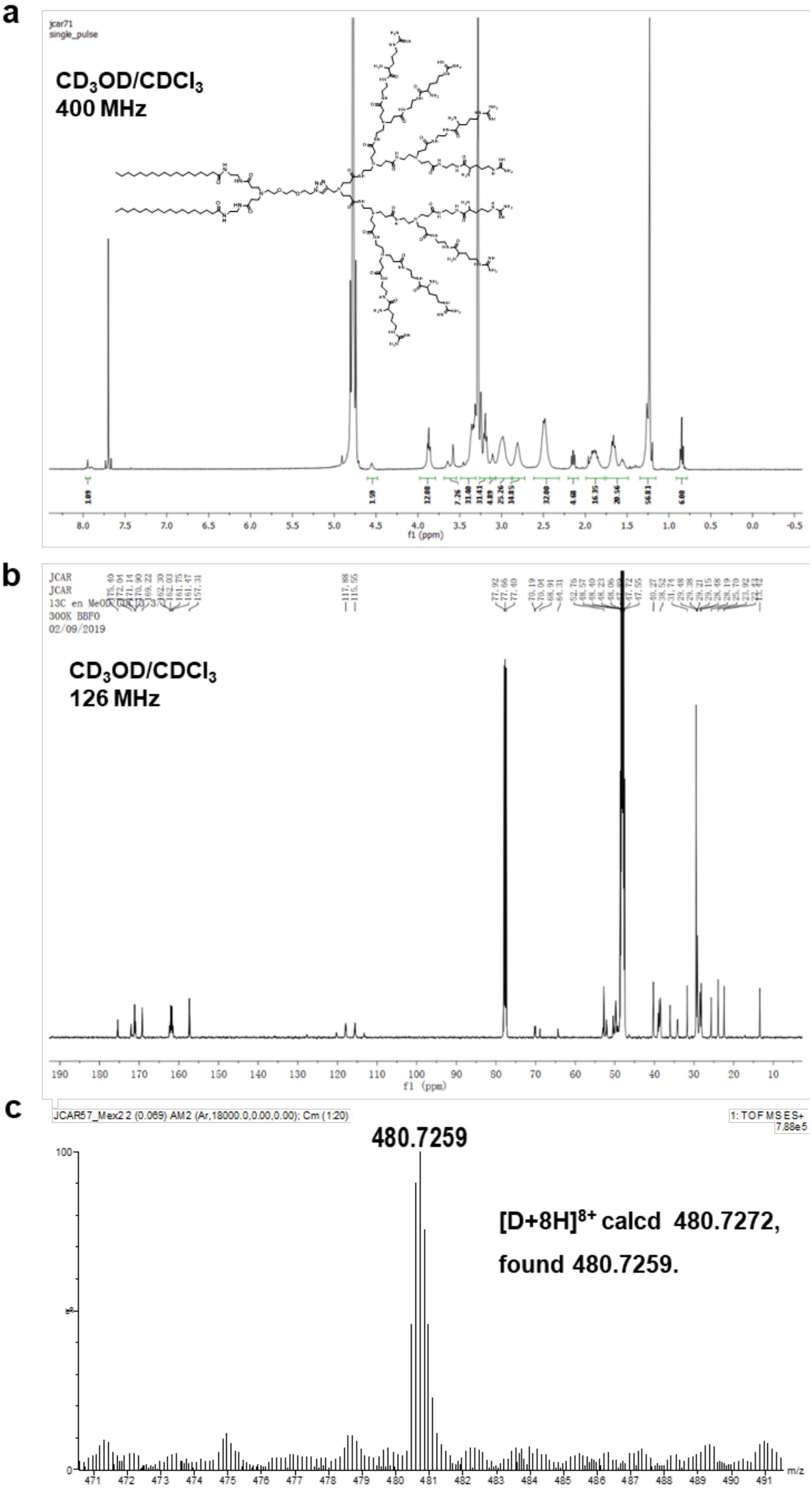

Fig. S3. Biodistribution of the Cy5-labeled RNA/dendrimer complexes in tumor and major organs of PDAC079T tumor xenograft mice determined using ex vivo fluorescence imaging.

(a) fluorescent images and (b) quantitative analysis of fluorescence signals of tumors and major organs resected from PDAC079T xenograft mice after intravenous administration of Cy5-labeled scramble RNA/**D**. Data: mean ± SD (n=3). Significance by one-way ANOVA/Tukey: *$P < 0.05$; **$P < 0.01$.

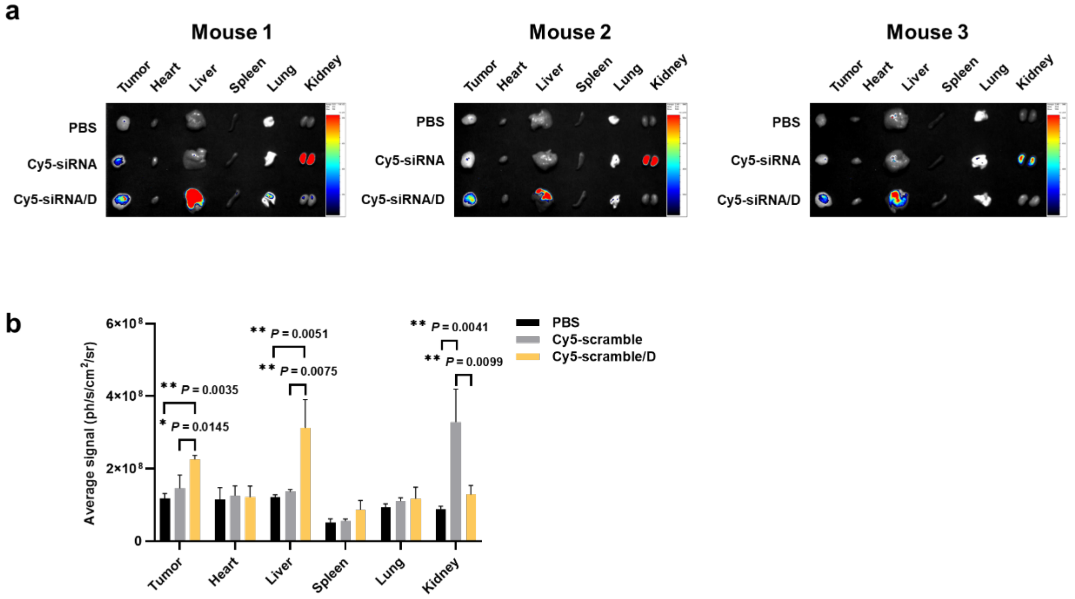

Fig. S4. Comparison of two preparation strategies for siRNA/saRNA combination delivery systems.

PDAC087T cells treated with non-treatment (control), siBIRC5/D, saCDKN1A/D, siBIRC5/D+saCDKN1A/D or (siBIRC5+saCDKN1A)/D, and the consequent regulation of *BIRC5* and *CDKN1A* at (a) mRNA level, (b) protein expression level, and (c) cell proliferation. RNA concentration: 25 nM; N/P ratio at 10. After 48 hours treatment, mRNA expression was quantified using qRT-PCR. After 72 hours treatment, protein expression was assessed using western blotting. At 5 days post-treatment, cell proliferation was evaluated using the MTT assay. Data: mean ± SD (n=3). Significance by one-way ANOVA/Tukey: ns not significant; *$P < 0.05$, **$P < 0.01$, ***$P < 0.001$, ****$P < 0.0001$.

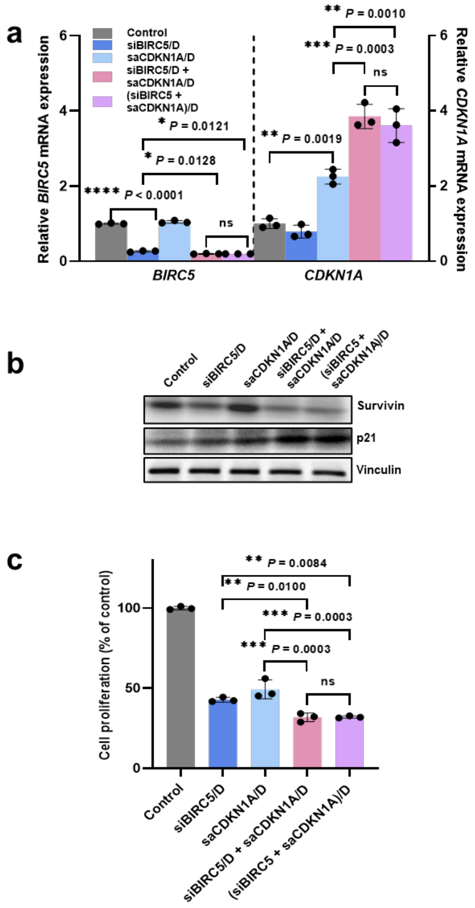

Fig. S5. Evaluation of the siRNA/saRNA combinations in primary PDC models for gene regulation and the consequential antiproliferative activity.

(a-c) PDAC087T cells treated with non-treatment (control), siMYC/D, saCEBPA/D, or combination: expression of *MYC* and *CEBPA* at (A) mRNA level and (b) protein level, and (c) cell proliferation. (d-f) PDAC079T cells treated with non-treatment (control), siAKT2/D, saCEBPA/D, or combination: expression of *AKT2* and *CEBPA* at (d) mRNA level and (e) protein level, and (f) cell proliferation. (g-i) PDAC079T cells treated with non-treatment (control), siMYC/D, saCDKN1A/D, or combination: expression of *MYC* and *CDKN1A* at (g) mRNA level and (h) protein level, and (i) cell proliferation. (j-l) PDAC036T cells treated with non-treatment (control), siBIRC5/D, saCDKN1A/D, or combination: expression of *BIRC5* and *CDKN1A* at (j) mRNA level and (k) protein level, and (l) cell proliferation (MTT at 5 days post-treatment). (m-o) PDAC036T cells treated with non-treatment (control), siBIRC5/D, saCEBPA/D, or combination: expression of *BIRC5* and *CEBPA* at (m) mRNA level and (n) protein level, and (o) cell proliferation (MTT at 5 days post-treatment). (p-r) PDAC084T cells treated with non-treatment (control), siMYC/D, saCEBPA/D, or combination: expression of *MYC* and *CEBPA* at (p) mRNA level and (q) protein level, and (r) cell proliferation (MTT at 5 days post-treatment). (s-u) PDAC084T cells treated with non-treatment (control), siBIRC5/D, saCDKN1A/D, or combination: expression of *BIRC5* and *CDKN1A* at (s) mRNA level and (t) protein level, and (u) cell proliferation (MTT at 5 days post-treatment). (v-x) PDAC082T cells treated with non-treatment (control), siBIRC5/D, saCDKN1A/D, or combination: expression of *BIRC5* and *CDKN1A* at (v) mRNA level and (w) protein level, and (x) cell proliferation (MTT at 5 days post-treatment). (y-aa) PDAC082T cells treated with non-treatment (control), siMYC/D, saCDKN1A/D, or combination: expression of *MYC* and *CDKN1A* at (y) mRNA level and (z) protein level, and (aa) cell proliferation (MTT at 5 days post-treatment). After 48 hours treatment, mRNA expression was quantified using qRT-PCR. After 72 hours treatment, protein expression was assessed using western blotting. At 5 days post-treatment, cell proliferation was evaluated using the MTT assay. Data: mean ± SD (n=3). Significance by one-way ANOVA/Tukey: *$P < 0.05$, **$P < 0.01$, ***$P < 0.001$, ****$P < 0.0001$.

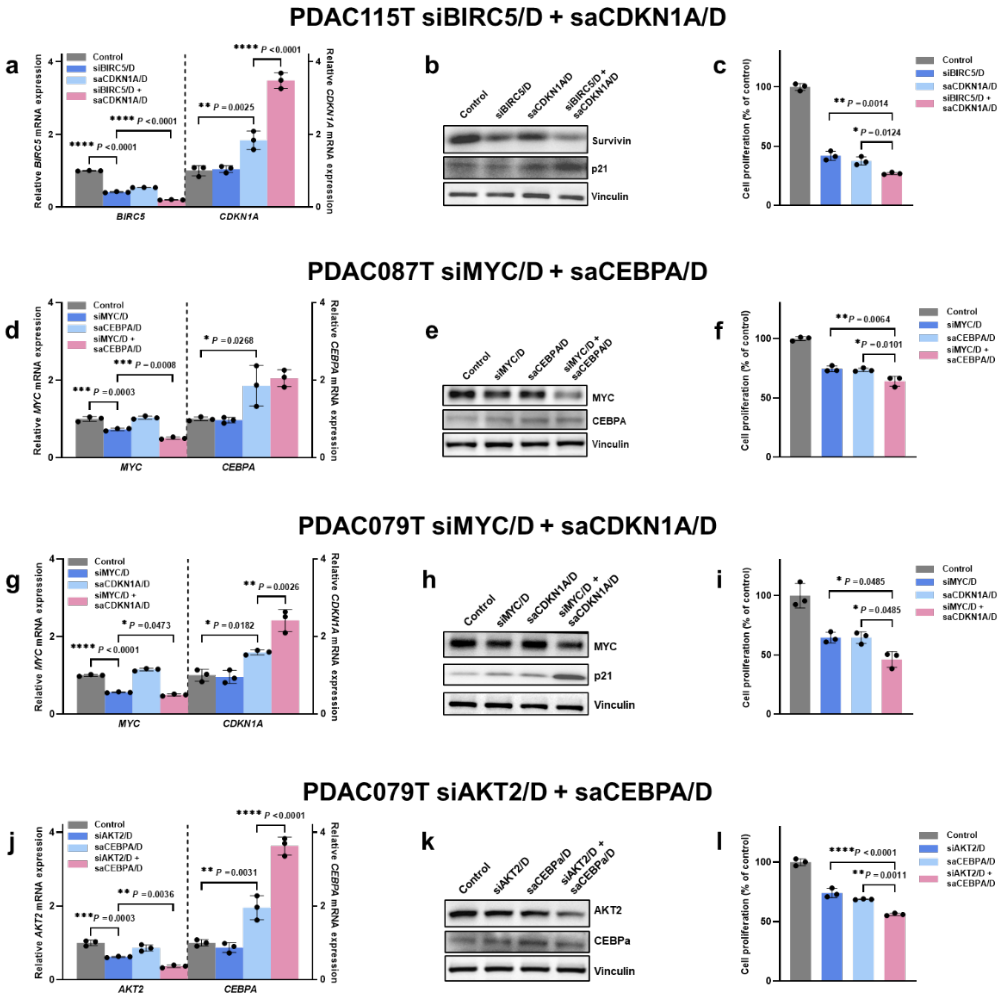

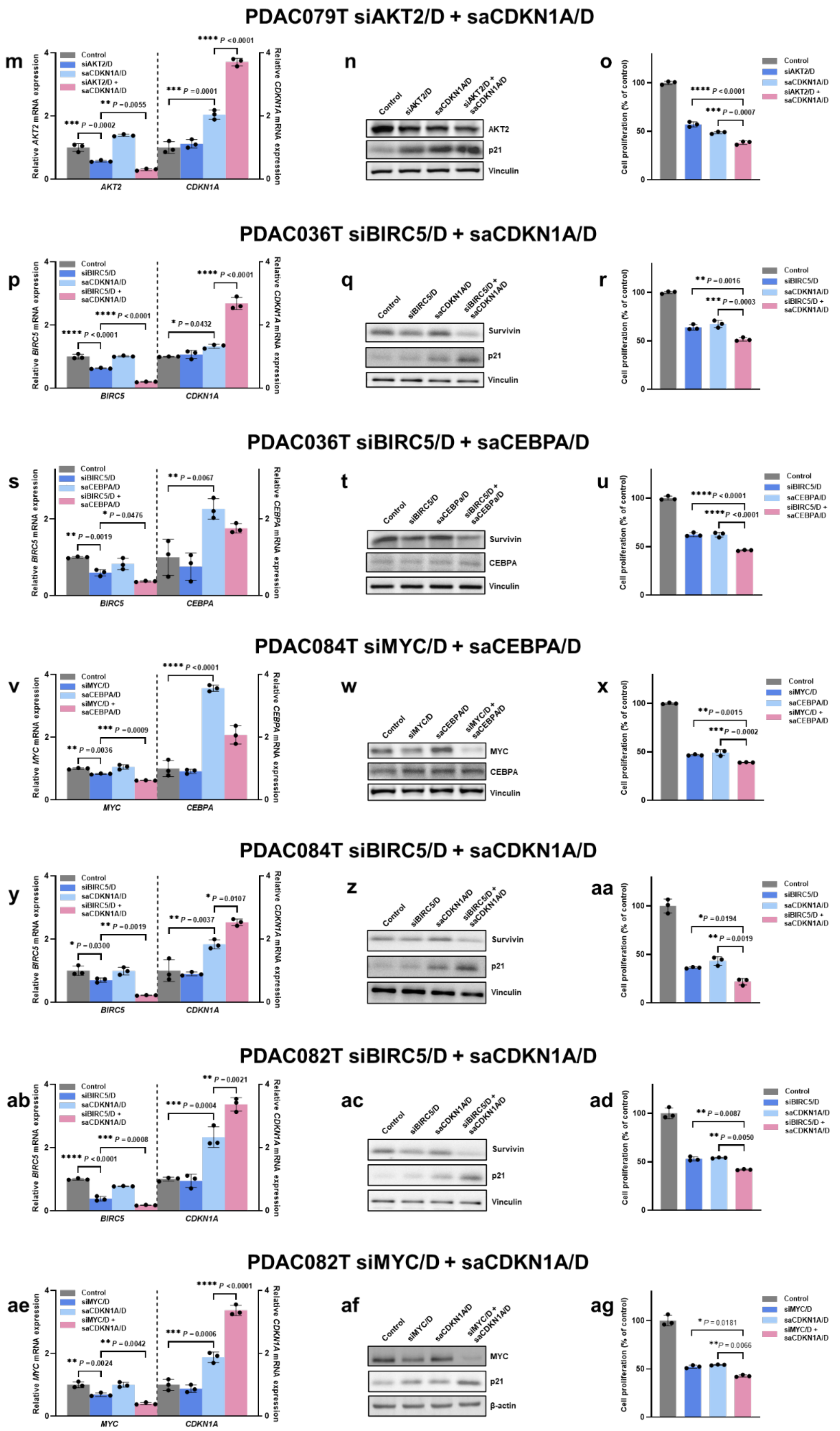
PDAC079T siAKT2/D + saCDKN1A/D
m
n
o
Control
siAKT2/D
saCDKN1A/D
siAKT2/D + saCDKN1A/D
AKT2
p21
Vinculin
Relative AKT2 mRNA expression
Relative CDKN1A mRNA expression
Cell proliferation (% of control)
PDAC036T siBIRC5/D + saCDKN1A/D
p
q
r
siBIRC5/D
siBIRC5/D + saCDKN1A/D
Survivin
Relative BIRC5 mRNA expression
PDAC036T siBIRC5/D + saCEBPA/D
s
t
u
saCEBPA/D
siBIRC5/D + saCEBPA/D
CEBPA
Relative CEBPA mRNA expression
PDAC084T siMYC/D + saCEBPA/D
v
w
x
siMYC/D
siMYC/D + saCEBPA/D
MYC
Relative MYC mRNA expression
PDAC084T siBIRC5/D + saCDKN1A/D
y
z
aa
PDAC082T siBIRC5/D + saCDKN1A/D
ab
ac
ad
PDAC082T siMYC/D + saCDKN1A/D
ae
af
ag
siMYC/D + saCDKN1A/D
β-actin

Fig. S6. Synergy score heatmaps of saRNA/D and siRNA/D combinations in PDCs.

Synergy scores of saRNA/D and siRNA/D combinations were calculated based on cell viability results using the HSA model implemented in SynergyFinder. Synergy score > 10: synergistic effect; Synergy score between −10 and 10: additive effect; Synergy score < -10: antagonistic effect. The value of mean synergy score was also provided for each combination.

(a) siAKT2/D+saCDKN1A/D in PDAC115T. (b) siMYC/D+saCDKN1A/D in PDAC087T. (c) siBIRC5/D+saCDKN1A/D in PDAC087T. (d) siBIRC5/D+saCDKN1A/D in PDAC079T. (e) siBIRC5/D+saCDKN1A/D in PDAC115T. (f) siMYC/D+saCEBPA/D in PDAC087T. (g) siMYC/D+saCDKN1A/D in PDAC079T. (h) siAKT2/D+saCEBPA/D in PDAC079T. (i) siBIRC5/D+saCEBPA/D in PDAC079T. (j) siMYC/D+saCEBPA/D in PDAC036T. (k) siAKT2/D+saCDKN1A/D in PDAC079T. (l) siBIRC5/D+saCDKN1A/D in PDAC036T. (m) siBIRC5/D+saCEBPA/D in PDAC036T. (n) siBIRC5/D+saCDKN1A/D in PDAC084T. (o) siMYC/D+saCEBPA/D in PDAC084T. (p) siBIRC5/D+saCDKN1A/D in PDAC082T. (q) siMYC/D+saCDKN1A/D in PDAC082T.

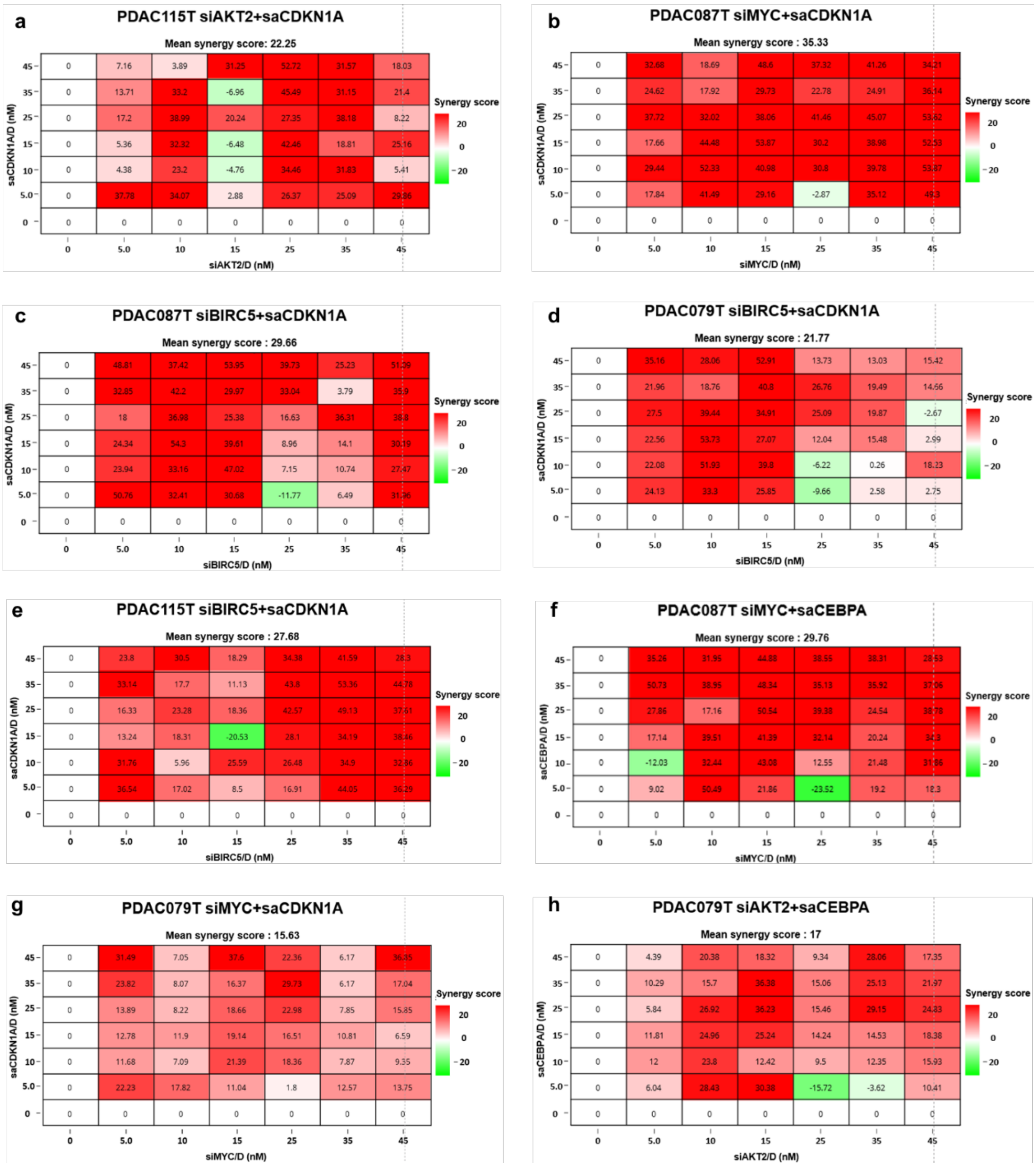

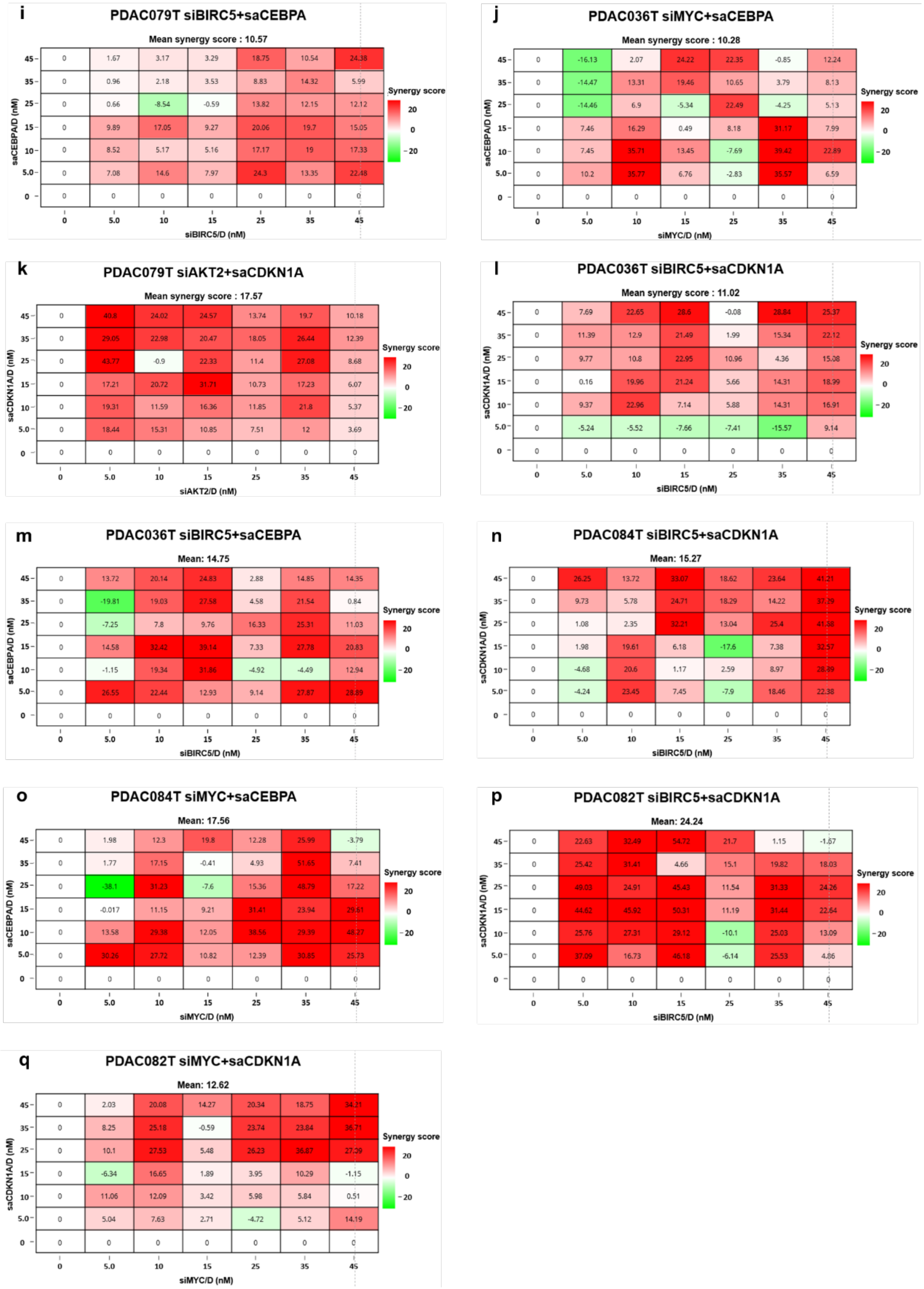
i
PDAC079T siBIRC5+saCEBPA
Mean synergy score : 10.57
saCEBPA/D (nM)
siBIRC5/D (nM)
Synergy score
j
PDAC036T siMYC+saCEBPA
Mean synergy score : 10.28
saCEBPA/D (nM)
siMYC/D (nM)
Synergy score
k
PDAC079T siAKT2+saCDKN1A
Mean synergy score : 17.57
saCDKN1A/D (nM)
siAKT2/D (nM)
Synergy score
l
PDAC036T siBIRC5+saCDKN1A
Mean synergy score : 11.02
saCDKN1A/D (nM)
siBIRC5/D (nM)
Synergy score
m
PDAC036T siBIRC5+saCEBPA
Mean: 14.75
saCEBPA/D (nM)
siBIRC5/D (nM)
Synergy score
n
PDAC084T siBIRC5+saCDKN1A
Mean: 15.27
saCDKN1A/D (nM)
siBIRC5/D (nM)
Synergy score
o
PDAC084T siMYC+saCEBPA
Mean: 17.56
saCEBPA/D (nM)
siMYC/D (nM)
Synergy score
p
PDAC082T siBIRC5+saCDKN1A
Mean: 24.24
saCDKN1A/D (nM)
siBIRC5/D (nM)
Synergy score
q
PDAC082T siMYC+saCDKN1A
Mean: 12.62
saCDKN1A/D (nM)
siMYC/D (nM)
Synergy score

Fig. S7. Evaluation of the siRNA/saRNA combinations in PDO models for gene regulation and organoid growth.

(a-d) PDAC087T organoids treated with non-treatment (control), siMYC/D, saCEBPA/D, or siMYC/D+saCEBPA/D combination: (a) Protein expression of MYC and CEBPA. (b) Organoid viability. (c) Organoid morphology. (d) Organoid diameter quantification from (c). (e-h) PDAC079T organoids treated with non-treatment (control), siAKT2/D, saCEBPA/D, or siAKT2/D+saCEBPA/D combination: (e) Protein expression of AKT2 and CEBPA. (f) Organoid viability. (g) Organoid morphology. (h) Organoid diameter quantification from (g). (i-l) PDAC036T organoids treated with non-treatment (control), siBIRC5/D, saCEBPA/D, or siBIRC5/D+saCEBPA/D combination: (i) Protein expression of Survivin and CEBPA. (j) Organoid viability. (k) Organoid morphology. (l) Organoid diameter quantification from (k). (m-p) PDAC036T organoids treated with non-treatment (control), siBIRC5/D, saCDKN1A/D, or siBIRC5/D+saCDKN1A/D combination: (m) Protein expression of Survivin and p21. (n) Organoid viability. (o) Organoid morphology. (p) Organoid diameter quantification from (o). (q-t) PDAC084T organoids treated with non-treatmentO (control), siMYC/D, saCEBPA/D, or siMYC/D+saCEBPA/D combination: (q) Protein expression of MYC and CEBPA. (r) Organoid viability. (s) Organoid morphology. (t) Organoid diameter quantification from (s). After 72 h treatment, protein expression was assessed using western blotting. At 7 days post-treatment, organoid viability was evaluated using ATP-based viability assay and organoid morphology was assessed using light microscopy. Data: mean ± SD (n=3). Significance by one-way ANOVA/Tukey: *$P < 0.05$, **$P < 0.01$, ***$P < 0.001$, ****$P < 0.0001$. Scale bar: 650 μm.

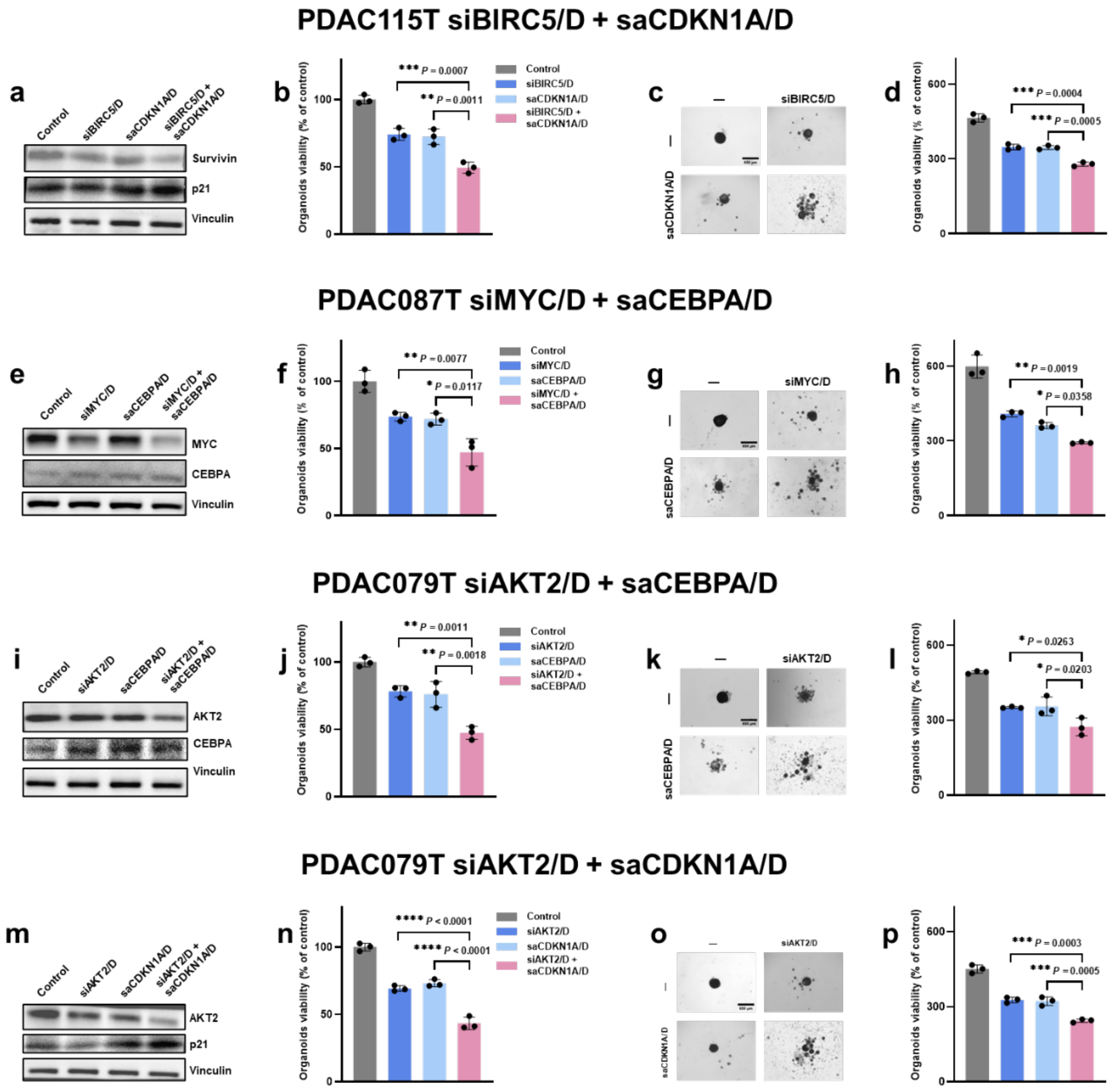

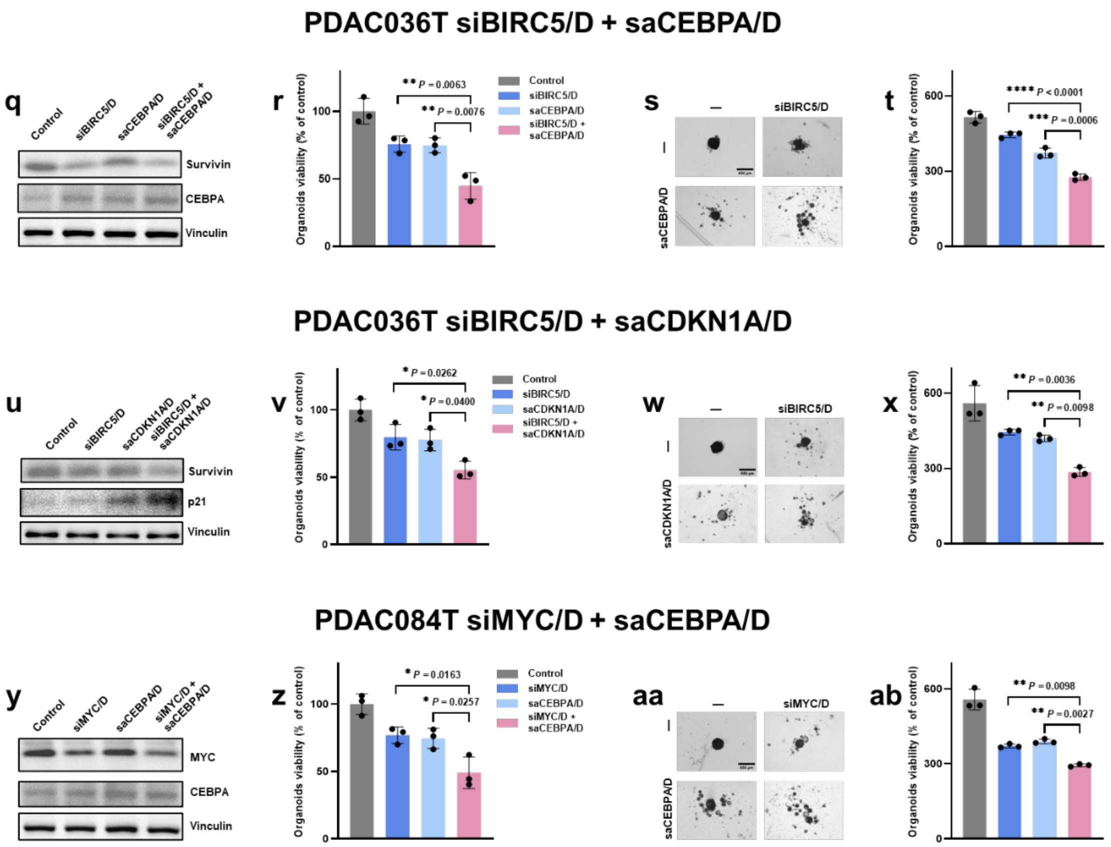
PDAC036T siBIRC5/D + saCEBPA/D
q
Control
siBIRC5/D
saCEBPA/D
siBIRC5/D + saCEBPA/D
Survivin
CEBPA
Vinculin
r
Organoids viability (% of control)
** P = 0.0063
** P = 0.0076
Control
siBIRC5/D
saCEBPA/D
siBIRC5/D + saCEBPA/D
s
—
siBIRC5/D
saCEBPA/D
t
**** P < 0.0001
*** P = 0.0006
PDAC036T siBIRC5/D + saCDKN1A/D
u
Control
siBIRC5/D
saCDKN1A/D
siBIRC5/D + saCDKN1A/D
Survivin
p21
Vinculin
v
* P = 0.0262
* P = 0.0400
Control
siBIRC5/D
saCDKN1A/D
siBIRC5/D + saCDKN1A/D
w
—
siBIRC5/D
saCDKN1A/D
x
** P = 0.0036
** P = 0.0098
PDAC084T siMYC/D + saCEBPA/D
y
Control
siMYC/D
saCEBPA/D
siMYC/D + saCEBPA/D
MYC
CEBPA
Vinculin
z
* P = 0.0163
* P = 0.0257
Control
siMYC/D
saCEBPA/D
siMYC/D + saCEBPA/D
aa
—
siMYC/D
saCEBPA/D
ab
** P = 0.0098
** P = 0.0027

Fig. S8. Evaluation of tumor cell proliferation and apoptosis in PDX models after treatment with siRNA/saRNA combinations.

(a) Proliferation (Ki67) and apoptosis (TUNEL, cleaved caspase-3) of PDAC115T PDX tumors following treatment with PBS (control), siAKT2/D (0.2 mg/kg siAKT2), saCDKN1A/D (0.2 mg/kg saCDKN1A), or combination (0.2 mg/kg each RNA). (b) Proliferation (Ki67) and apoptosis (TUNEL, cleaved caspase-3) of PDAC087T PDX mice tumors following treatment with PBS (control), D alone, siMYC alone, siMYC/D (0.1 mg/kg siMYC), saCDKN1A/D (0.1 mg/kg saCDKN1A), or combination (0.1 mg/kg each RNA). (c) Proliferation (Ki67) and apoptosis (TUNEL, cleaved caspase-3) of PDAC087T PDX mice tumors following treatment with PBS (control), D alone, siBIRC5 alone, siBIRC5/D (0.1 mg/kg siBIRC5), saCDKN1A/D (0.1 mg/kg saCDKN1A), or combination (0.1 mg/kg each RNA). (d) Proliferation (Ki67) and apoptosis (TUNEL, cleaved caspase-3) of PDAC079T PDX mice tumors following treatment with PBS (control), siBIRC5/D (0.2 mg/kg siBIRC5), saCDKN1A/D (0.2 mg/kg saCDKN1A), or combination (0.1 or 0.2 mg/kg each RNA). (e) Proliferation (Ki67) and apoptosis (TUNEL, cleaved caspase-3) of PDAC079T PDX mice tumors following treatment with PBS (control), siBIRC5/D (0.2 mg/kg siBIRC5), saCEBPA/D (0.2 mg/kg saCEBPA), or combination (0.1 or 0.2 mg/kg each RNA). (f) Proliferation (Ki67) and apoptosis (TUNEL, cleaved caspase-3) of PDAC036T PDX mice tumors following treatment with PBS (control), siMYC/D (0.2 mg/kg siMYC), saCEBPA/D (0.2 mg/kg saCEBPA), or combination (0.1 or 0.2 mg/kg each RNA). Scale bars: 200 µm.

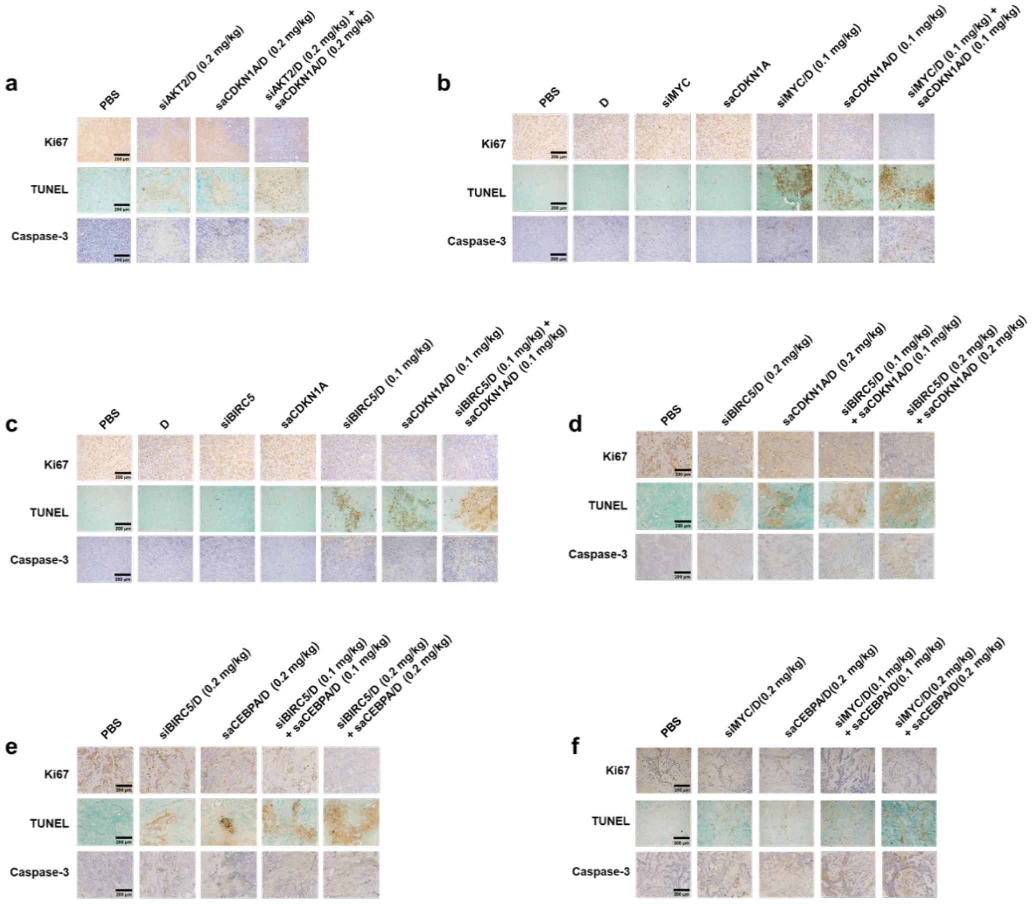

Fig. S9. Evaluation of PDX mice health following treatment with siRNA/saRNA combinations.

(a) Body weight and (b) H&E staining of main organs issued from PDAC115T PDX mice treated with PBS (control), siAKT2/D (0.2 mg/kg siAKT2), saCDKN1A/D (0.2 mg/kg saCDKN1A), or combination (0.2 mg/kg each RNA). (c) Body weight and (d) H&E staining of main organs issued from PDAC087T PDX mice treated with PBS (control), D alone, siMYC alone, siMYC/D (0.1 mg/kg siMYC), saCDKN1A/D (0.1 mg/kg saCDKN1A), or combination (0.1 mg/kg each RNA). (e) Body weight and (f) H&E staining of main organs issued from PDAC087T PDX mice treated with PBS (control), D alone, siBIRC5 alone, siBIRC5/D (0.1 mg/kg siBIRC5), saCDKN1A/D (0.1 mg/kg saCDKN1A), or combination (0.1 mg/kg each RNA). (g) Body weight and (h) H&E staining of main organs issued from PDAC079T PDX mice treated with PBS (control), siBIRC5/D (0.2 mg/kg siBIRC5), saCDKN1A/D (0.2 mg/kg saCDKN1A), or combination (0.1 or 0.2 mg/kg each RNA). (i) Body weight and (j) H&E staining of main organs issued from PDAC079T PDX mice treated with PBS (control), siBIRC5/D (0.2 mg/kg siBIRC5), saCEBPA/D (0.2 mg/kg saCEBPA), or combination (0.1 or 0.2 mg/kg each RNA). (k) Body weight and (l) H&E staining of main organs issued from PDAC036T PDX mice treated with PBS (control), siMYC/D (0.2 mg/kg siMYC), saCEBPA/D (0.2 mg/kg saCEBPA), or combination (0.1 or 0.2 mg/kg each RNA). Data: mean ± SEM (n=4/5). Scale bars: 200 µm.

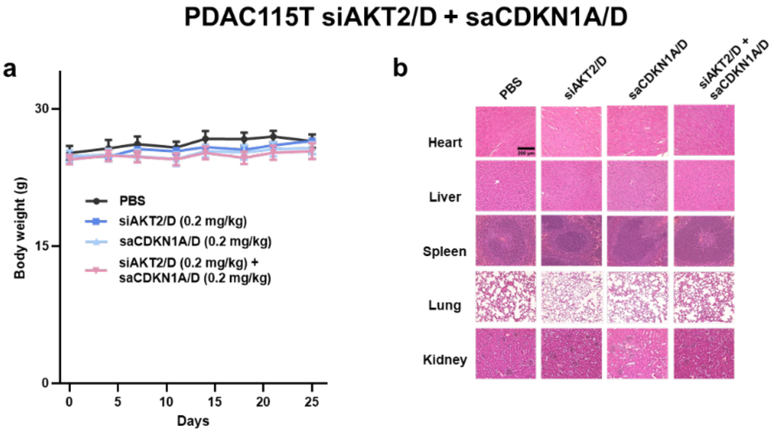

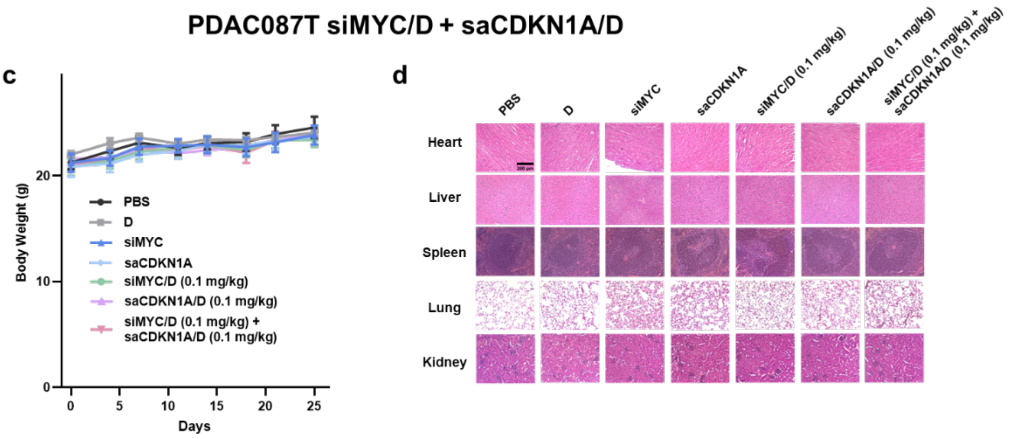

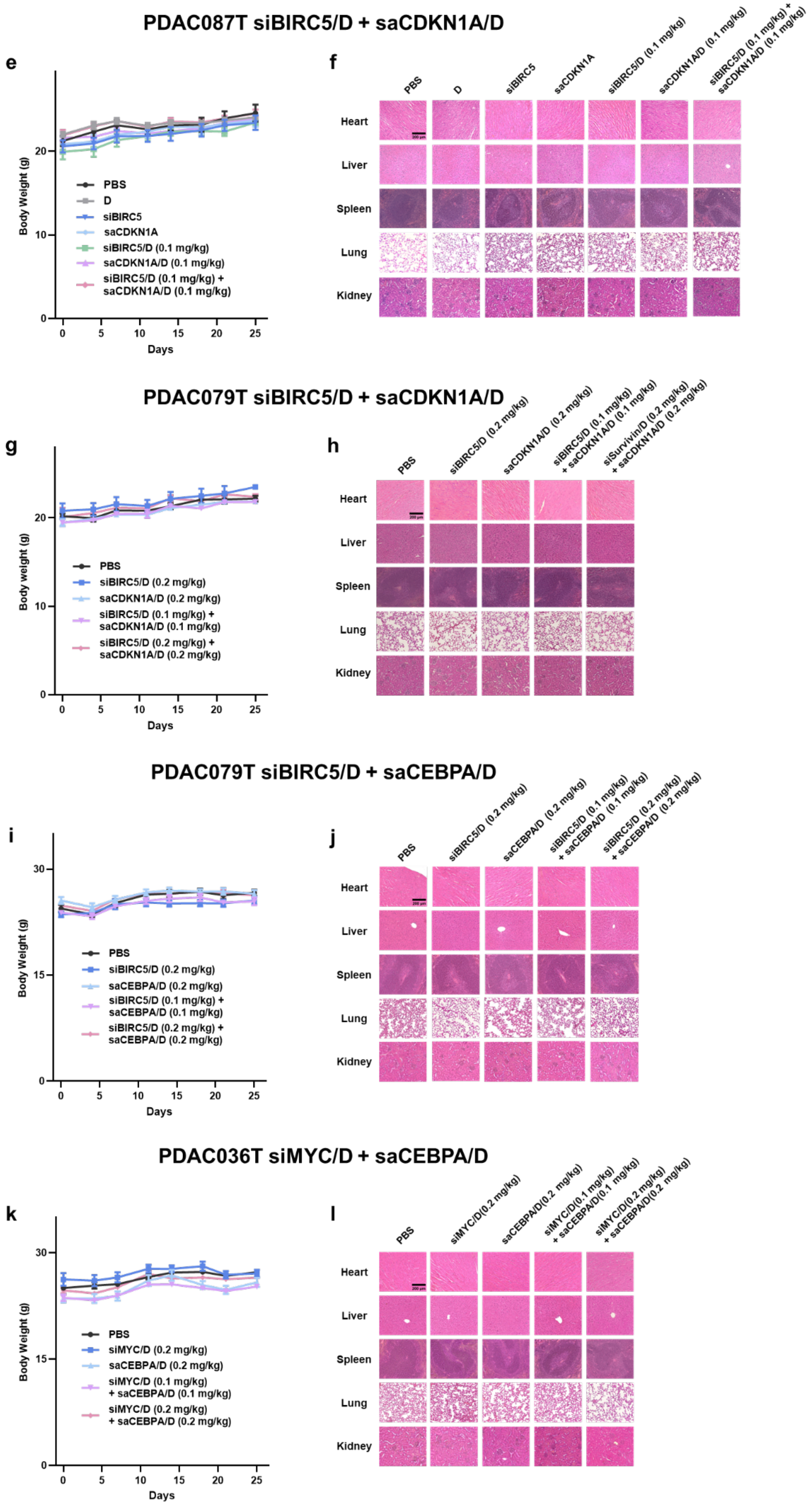
PDAC087T siBIRC5/D + saCDKN1A/D
e
Body Weight (g)
Days
PBS
D
siBIRC5
saCDKN1A
siBIRC5/D (0.1 mg/kg)
saCDKN1A/D (0.1 mg/kg)
siBIRC5/D (0.1 mg/kg) + saCDKN1A/D (0.1 mg/kg)
f
PBS
D
siBIRC5
saCDKN1A
siBIRC5/D (0.1 mg/kg)
saCDKN1A/D (0.1 mg/kg)
siBIRC5/D (0.1 mg/kg) + saCDKN1A/D (0.1 mg/kg)
Heart
Liver
Spleen
Lung
Kidney
PDAC079T siBIRC5/D + saCDKN1A/D
g
Body weight (g)
Days
PBS
siBIRC5/D (0.2 mg/kg)
saCDKN1A/D (0.2 mg/kg)
siBIRC5/D (0.1 mg/kg) + saCDKN1A/D (0.1 mg/kg)
siBIRC5/D (0.2 mg/kg) + saCDKN1A/D (0.2 mg/kg)
h
PBS
siBIRC5/D (0.2 mg/kg)
saCDKN1A/D (0.2 mg/kg)
siBIRC5/D (0.1 mg/kg) + saCDKN1A/D (0.1 mg/kg)
siSurvivin/D (0.2 mg/kg) + saCDKN1A/D (0.2 mg/kg)
Heart
Liver
Spleen
Lung
Kidney
PDAC079T siBIRC5/D + saCEBPA/D
i
Body Weight (g)
Days
PBS
siBIRC5/D (0.2 mg/kg)
saCEBPA/D (0.2 mg/kg)
siBIRC5/D (0.1 mg/kg) + saCEBPA/D (0.1 mg/kg)
siBIRC5/D (0.2 mg/kg) + saCEBPA/D (0.2 mg/kg)
j
PBS
siBIRC5/D (0.2 mg/kg)
saCEBPA/D (0.2 mg/kg)
siBIRC5/D (0.1 mg/kg) + saCEBPA/D (0.1 mg/kg)
siBIRC5/D (0.2 mg/kg) + saCEBPA/D (0.2 mg/kg)
Heart
Liver
Spleen
Lung
Kidney
PDAC036T siMYC/D + saCEBPA/D
k
Body Weight (g)
Days
PBS
siMYC/D (0.2 mg/kg)
saCEBPA/D (0.2 mg/kg)
siMYC/D (0.1 mg/kg) + saCEBPA/D (0.1 mg/kg)
siMYC/D (0.2 mg/kg) + saCEBPA/D (0.2 mg/kg)
l
PBS
siMYC/D(0.2 mg/kg)
saCEBPA/D(0.2 mg/kg)
siMYC/D(0.1 mg/kg) + saCEBPA/D(0.1 mg/kg)
siMYC/D(0.2 mg/kg) + saCEBPA/D(0.2 mg/kg)
Heart
Liver
Spleen
Lung
Kidney

Table S1. The sequences of saRNAs and siRNAs used in this study

siRNA/saRNA	Sequences	
	Sense (5'-3')	Antisense (5'-3')
siAKT2	GCUCCUUCAUUGGGUACAAdTdT	UUGUACCCAAUGAAGGAGCdTdT
siMYC	CAAGGUAGUUAUCCUUAAAAAAGCCACdTdT	GUGGCUUUUUUAAGGAUAACUACCUUGdTdT
siBIRC5	GAAUUUGAGGAAACUGCGAdTdT	UCGCAGUUUCCUCAAAUUCdTdT
saCDKN1A	CCAACUCAUUCUCCAAGUAdTdT	UACUUGGAGAAUGAGUUGGdTdT
saCEBPA	GCGGUCAUUGUCACUGGUCdTdT	GACCAGUGACAAUGACCGCdTdT

Table S2. Synergistic effects of the saRNA/D and /siRNA/D combinations in PDAC PDCs. Combination index (*CI*) was calculated based on the cell proliferation results presented in Figure 4 and Figure S5 using Highest Single Agent (HSA) model. *E* represents the inhibition effect on cell proliferation. *CI* < 1 was considered synergistic effect, *CI* = 1 was considered additive effect, and *CI* > 1 was considered antagonistic effect.

PDC: siRNA/saRNA combination	$E_{siRNA/D}$	$E_{saRNA/D}$	$E_{(siRNA/D+saRNA/D)}$	CI_{HSA}
PDAC115T (siAKT2/D+saCDKN1A/D)	0.60	0.61	0.72	0.84
PDAC087T (siMYC/D+saCDKN1A/D)	0.38	0.33	0.68	0.56
PDAC087T (siBIRC5/D+saCDKN1A/D)	0.35	0.33	0.58	0.61
PDAC079T (siBIRC5/D+saCDKN1A/D)	0.37	0.35	0.54	0.68
PDAC079T (siBIRC5/D+saCEBPA/D)	0.24	0.31	0.40	0.78
PDAC036T (siMYC/D+saCEBPA/D)	0.42	0.38	0.55	0.77
PDAC115T (siBIRC5/D+saCDKN1A/D)	0.58	0.63	0.73	0.86
PDAC087T (siMYC/D+saCEBPA/D)	0.26	0.26	0.36	0.73
PDAC079T (siMYC/D+saCDKN1A/D)	0.35	0.36	0.54	0.66
PDAC079T (siAKT2/D+saCEBPA/D)	0.26	0.31	0.44	0.71
PDAC079T (siAKT2/D+saCDKN1A/D)	0.43	0.51	0.62	0.82
PDAC036T (siBIRC5/D+saCDKN1A/D)	0.36	0.33	0.48	0.74
PDAC036T (siBIRC5/D+saCEBPA/D)	0.38	0.38	0.54	0.70
PDAC084T (siMYC/D+saCEBPA/D)	0.53	0.51	0.61	0.87
PDAC084T (siBIRC5/D+saCDKN1A/D)	0.64	0.57	0.78	0.82
PDAC082T (siBIRC5/D+saCDKN1A/D)	0.47	0.46	0.58	0.81
PDAC082T (siMYC/D+saCDKN1A/D)	0.48	0.46	0.57	0.85

Table S3: Synergistic effects of the saRNA/D and siRNA/D combinations in PDOs. Combination index (*CI*) was calculated based on the organoid viability results presented in Figure 5 and Figure S7 using both Highest Single Agent (HSA) and Bliss Independence (Bliss) models. *E* represents the inhibition effect on organoid viability. *CI* < 1 was considered synergistic effect, *CI* = 1 was considered additive effect, and *CI* > 1 was considered antagonistic effect.

PDO: siRNA/saRNA combination	$E_{siRNA/D}$	$E_{saRNA/D}$	$E_{(siRNA/D+saRNA/D)}$	CI_{HSA}	CI_{Bliss}
PDAC115T (siAKT2+saCDKN1A)	0.25	0.32	0.52	0.62	0.95
PDAC087T (siMYC+saCDKN1A)	0.20	0.20	0.53	0.38	0.67
PDAC087T (siBIRC5+saCDKN1A)	0.29	0.27	0.55	0.52	0.86
PDAC079T (siBIRC5+saCDKN1A)	0.29	0.23	0.51	0.57	0.89
PDAC079T (siBIRC5+saCEBPA)	0.24	0.19	0.51	0.48	0.76
PDAC036T (siMYC+saCEBPA)	0.34	0.38	0.61	0.63	0.97
PDAC115T (siBIRC5+saCDKN1A)	0.26	0.28	0.51	0.54	0.91
PDAC087T (siMYC+saCEBPA)	0.26	0.28	0.53	0.53	0.89
PDAC079T (siAKT2+saCEBPA)	0.22	0.24	0.53	0.46	0.77
PDAC079T (siAKT2+saCDKN1A)	0.31	0.27	0.57	0.54	0.87
PDAC036T (siBIRC5+saCEBPA)	0.24	0.25	0.55	0.46	0.79
PDAC036T (siBIRC5+saCDKN1A)	0.20	0.22	0.45	0.50	0.85
PDAC084T (siMYC+saCEBPA)	0.23	0.25	0.51	0.50	0.84

Table S4: Synergistic effects of the saRNA/D and siRNA/D combinations in PDXs. Combination index (*CI*) was calculated based on the tumor volume results presented in Figure 6 using both HSA and Bliss models. *E* represents the inhibition on tumor volume relative to the PBS group. *CI* < 1 was considered synergistic effect, *CI* = 1 was considered additive effect, and *CI* > 1 was considered antagonistic effect.

PDX: siRNA/saRNA combination	$E_{siRNA/D}$	$E_{saRNA/D}$	$E_{(siRNA/D+saRNA/D)}$	CI_{HSA}	CI_{Bliss}
PDAC115T (siAKT2+saCDKN1A)	0.56	0.55	0.93	0.61	0.87
PDAC087T (siMYC+saCDKN1A)	0.53	0.54	0.79	0.69	0.99
PDAC087T (siBIRC5+saCDKN1A)	0.52	0.54	0.80	0.68	0.98
PDAC079T (siBIRC5+saCDKN1A) (0.10 mg/kg+0.10 mg/kg)	0.24	0.29	0.58	0.51	0.80
PDAC079T (siBIRC5+saCDKN1A) (0.20 mg/kg+0.20 mg/kg)	0.24	0.29	0.77	0.38	0.60
PDAC079T (siBIRC5+saCEBPA) (0.10 mg/kg+0.10 mg/kg)	0.38	0.38	0.62	0.61	0.99
PDAC079T (siBIRC5+saCEBPA) (0.20 mg/kg+0.20 mg/kg)	0.38	0.38	0.80	0.47	0.76
PDAC036T (siMYC+saCEBPA) (0.10 mg/kg+0.10 mg/kg)	0.45	0.40	0.84	0.53	0.79
PDAC036T (siMYC+saCEBPA) (0.20 mg/kg+0.20 mg/kg)	0.45	0.40	0.88	0.51	0.76

Materials and methods:

Dendrimer AD was synthesized according to protocols previously established in our group.[1, 2] Chemicals were purchased from Sigma Aldrich and were used without further purification. Dialysis tubing was purchased from Sigma Aldrich (St. Quentin Fallavier, France). 1H and ^{13}C-NMR spectra were recorded on Bruker Avance III 400 (400 MHz, ^{1}H) and Bruker Avance III 500 (125 MHz, ^{13}C). The temperature for all NMR data collection is 300 K if not specified. Chemical shifts (δ) are expressed in parts per million (ppm). The HRMS analysis was carried out with a SYNAPT G2 HDMS (Waters) mass spectrometer equipped with a pneumatically assisted atmospheric pressure ionization (API) source. High-resolution mass spectra (MS) were obtained with a flight time analyzer (TOF). Each accurate mass measurement was done in triplicate using an external calibration. All other siRNAs and saRNAs were purchased from GenePharma (China). The siRNAs and saRNAs sequence are lists in Table S1. All primers were synthesized by Eurofins Genomics (Germany).

Synthesis and characterization of the dendrimer D

To add Boc-Arg(Pbf)-OH (169 mg, 0.32 mmol), HOBt (43 mg, 0.32 mmol) and HBTU (121 mg, 0.32 mmol) into a flask then protect with Ar. Add DMF (2 mL) to dissolve the reagents followed by adding DIPEA (56 μL, 0.32 mmol) while stirring. Then a solution of AD (25 mg, 0.010 mmol) in DMF (2.5 mL) was added and the reaction was stirred for 72 h at 30 ºC under argon. Afterwards, the solvent was removed and the resulting residue was dissolved in 1 mL Methanol and precipitated by Ether. Repeat the precipitation for 3 times to give a white solid intermediate. To add TFA (4 mL), H_2O (0.12 mL) and Triisopropylsilane (0.12 mL) to the intermediate and the reaction solution was stirred at 30 ºC for 2 h. Then the TFA was removed and the obtained residue was purified by dialysis using dialysis tube of MWCO 2000, followed by lyophilization. After repeating 4 times the operation of dialysis and lyophilization, the product was lyophilized to give **D** (37 mg, 99%) as a white solid.^{1}H NMR (400 MHz, $CDCl_3/CD_3OD$) δ 7.95 (s, 1H), 4.55 (s, 2H), 3.87 (t, J = 6.2 Hz, 12H), 3.61 (d, J = 26.2 Hz, 6H), 3.34 (m, 52H), 3.26 – 3.14 (m, 16H), 3.11 (s, 4H), 2.98 (s, 28H), 2.81 (s, 14H), 2.49 (d, J = 6.5 Hz, 32H), 2.20 – 2.08 (m, 4H), 1.99 – 1.77 (m, 16H), 1.64 (dd, J = 28.0, 20.8 Hz, 20H), 1.35 – 1.16 (m, 56H), 0.84 (q, J = 6.3 Hz, 6H). ^{13}C NMR (126 MHz, $CDCl_3/CD_3OD$) δ 175.40, 172.04, 171.14, 170.90, 169.22, 162.30, 162.03, 161.75, 161.47, 157.31, 117.88, 115.55, 77.92, 77.66, 77.40, 70.19, 70.04, 68.91, 64.31, 53.00, 52.76, 52.07, 50.39, 49.76, 49.24, 48.57, 48.40, 48.23, 48.06, 47.89, 47.72, 47.55, 40.27, 39.08, 38.81, 38.52, 38.39, 36.06, 34.17, 31.74, 29.48, 29.38, 29.21, 29.15, 28.48, 28.19, 25.70, 23.92, 22.43, 13.42. HRMS m⁄z calcd for $C_{173}H_{343}N_{69}O_{28}$, $[M+8H]^{8+}$ 480.7272, found 480.7259.

Transmission electron microscopy (TEM)

Transmission electron microscopy (TEM) was conducted using a JEOL-JEM-2100F analytical electron microscope (Tokyo, Japan) to analyze the size and morphology of the nanoparticles at an accelerating voltage of 200 kV. The nanoparticle solutions were prepared in milliQ water at a concentration of 1.0 mg/mL, vortexed for 30 seconds, and then diluted to 15 μg/mL. A 4.0 μL aliquot of the solution was deposited onto a carbon-coated copper grid and place in a dark environment at 25 degrees for 15 minutes. Excess solution was removed using filter paper. The grid was then stained with 4.0 μL of uranyl acetate (2.0% aqueous solution) for 5 seconds, and any remaining uranyl acetate was removed with filter paper prior to imaging.

Dynamic light scattering (DLS) analysis

Complexes were prepared by mixing a solution of scrambled siRNA with a solution of dendrimer D in H_2O (pH adjusted to 7.4 using 0.10 M HCl) at N/P ratios of 10 or 5. The mixtures were incubated at 25 ° for 30 min prior to

analysis. The hydrodynamic size distribution and zeta potential were subsequently measured using a Zetasizer Nano ZS (Malvern Ltd., Malvern, UK) equipped with a 633 nm He–Ne laser. All measurements were performed in triplicate.

RNA/dendrimer complex formation

For in vitro transfection in cells and organoids

1.0 mL of a RNA/dendrimer complex was prepared as follows: the required amounts of RNA and dendrimer were each diluted in 0.10 mL of Opti-MEM transfection medium (Gibco), mixed gently by pipetting, and allowed to stand at room temperature for 10 minutes. Subsequently, the dendrimer solution was added to the nucleic acid solution, mixed thoroughly by pipetting for 10 seconds, and incubated at room temperature for an additional 30 minutes. The mixture was then supplemented with 0.80 mL of Opti-MEM medium to a final volume of 1.0 mL.

For in vivo transfection in tumor xenograft

A 0.20 mL RNA/dendrimer complex solution (RNA dose: 0.10 or 0.20 mg/kg) was prepared for each mouse as follows: the required amounts of RNA and dendrimer were each diluted in 0.10 mL of PBS. The two solutions were then gently mixed by pipetting and allowed to stand at room temperature for 10 min. Subsequently, the dendrimer solution was added to the RNA solution, followed by thorough mixing by pipetting for 10 s, and further incubated at room temperature for 30 min.

Cells

The MDCK, L929, CHO-K1, Raw264.7, and HEK293T cell lines were all purchased from the American Type Culture Collection (ATCC). CHO-K1 cell line was cultured in Roswell Park Memorial Institute Medium 1640 (RPMI 1640) supplemented with 10% (v/v) fetal bovine serum (FBS, Biosera), while the MDCK, L929, Raw264.7, and HEK293T cell lines were maintained in Dulbecco's Modified Eagle Medium (DMEM) supplemented with 10% (v/v) FBS. All cell lines were cultured at 37°C with 5% CO2 in a humidified incubator. The culture medium was changed every 2 to 3 days. Regular MYCoplasma testing was conducted to ensure the absence of contamination.

Preclinical PDAC models

Models used in this study are patient-derived primary cell cultures and patient-derived organoids obtained directly from patients tumors or from patient-derived xenografts.[3] The patients were included in the PaCaOmics clinical trial NCT01692873 (https:/clinicaltrials.gov/show/NCT01692873). Consent forms were collected from informed patients and recorded in a central database. The experimental procedure relating to the use of patient-derived pancreatic tumor models was performed after approval from the South Mediterranean Personal Protection Committee, under the reference 2011-A01439-32. Expert clinical centers collaborated on this project after approval from their respective ethics review board (approval number 11-61).

Primary patient-derived pancreatic cancer cells

Patient derived pancreatic cancer cells of PDAC087T, PDAC082T, PDAC084T, PDAC036T, PDAC079T and PDAC115T were obtained from PaCaOmics clinical trial. Cells were cultured in Serum-Free Ductal Media (SFDM), as adapted from Schreiber et al.,[4] SFDM was composed of DMEM/F12 medium supplemented with 1.22 g/L nicotinamide, 5.0 g/L glucose, 5.0% Nu-Serum IV, 0.50% ITS+ Premix Universal Culture Supplement (containing insulin, human transferrin, and selenous acid), 1.0 µM dexamethasone, 10 ng/L cholera toxin, 50 nM 3,3′,5-Triiodo-L-thyronine, 25.2 mg/L bovine pituitary extract, and 20 µg/L epidermal growth factor. All cell lines were cultured at 37°C with 5% CO_2 in a humidified incubator. The culture medium was changed every 2 to 3 days. Regular

MYCoplasma testing was conducted to ensure the absence of contamination. All primary patient-derived pancreatic cancer cells were maintained at 37°C in a humidified atmosphere containing 5% CO_2. Media were replaced every 2 to 3 days. Mycoplasma contamination was regularly monitored to ensure the cultures remained clean.

Patient-derived pancreatic cancer organoids

Patient derived pancreatic cancer organoids of PDAC087T, PDAC082T, PDAC084T, PDAC036T, PDAC079T and PDAC115T were obtained from PaCaOmics clinical trial. Organoids were placed into 12-well plates coated with 150 µL GFR Matrigel (Corning) and cultured with pancreatic organoid feeding media (POFM) consisting of advanced DMEM/F12 supplemented with 10 mM HEPES (Thermo Fisher), 1.0 × Glutamax (Thermo Fisher), penicillin/streptoMYCin (Thermo Fisher), 100 ng/mL animal-free recombinant human FGF10 (Peprotech), 50 ng/mL animal-free recombinant human EGF (Peprotech), 100 ng/mL recombinant human Noggin (Biotechne), Wnt3a-conditioned medium (30% v/v), RSPO1-conditioned medium (10% v/v), 10 nM human Gastrin 1 (Sigma Aldrich), 10 mM Nicotinamide (Sigma Aldrich), 1.25 mM N-acetylcysteine (Sigma Aldrich), 1.0 × B27 (Invitrogen), 500 nM A83-01 (Tocris), and 10.5 µM Y27632 (Tocris). Plates were incubated at 37°C in a 5% CO_2 atmosphere, with media changed every 3 to 4 days.

Cellular uptake

The uptake efficiency of the scramble RNA delivered by D and AD in PDAC087T cells were assessed using flow cytometry with the Cy3-labeled scramble RNA. The cells were seeded in a 24-well plate at 8.75×10^4 for 24 h, then incubated with Cy3-RNA/D and Cy3-RNA/AD complexes for 2h and 4h at 37°C. The cells were washed three times with cold PBS buffer and analyzed using a MACS® flow cytometry (Miltenyi Biotec, Miltenyi Biotec, Surrey, UK). Each assay was performed in triplicate. The data were analyzed using FlowJo software (V10).

MTT assay for dendrimer D cytotoxicity

HEK 293, L929, MDCK, CHO-K1, and Raw264.7 cells were seeded at a density of 4,000 cells per well in 100 µL of medium in 96-well plates and allowed to grow for 24 hours. The cells were then treated with D or scramble RNA/D complexes. After 8 hours of treatment, the transfection medium was replaced with complete medium containing 10% FBS, and the cells were incubated under normal growth conditions for an additional 48 hours. Next, MTT solution (5 mg/mL) was added to each well and incubated for 3 more hours. The obtained crystal was dissolved in DMSO, and the absorbance of the samples at 570 nm (OD_{570}) and the background absorbance at 690 nm (OD_{690}) were measured using a FLUOstar Omega microplate reader. The final true absorbance of the samples was calculated as $OD_{570} - OD_{690}$. Each measurement was performed in triplicate.

Lactate dehydrogenase (LDH) assay

HEK 293, L929, MDCK, CHO-K1, and RAW 264.7 cells were seeded at a density of 4,000 cells per well in 100 µL of medium in 96-well plates, 24 hours prior to treatment. The cells were then treated with D or scramble RNA/D for 8 hours at 37°C in a humidified atmosphere containing 5% CO_2. Following treatment, cell membrane integrity was assessed using the CytoTox-ONE™ Homogeneous Membrane Integrity Assay (Promega) according to the manufacturer's instructions. Prior to LDH assay, the 96-well plates were allowed to equilibrate to room temperature (22°C). Positive and negative controls were included by treating cells with lysis buffer and medium alone, corresponding to 100% and 0% LDH release, respectively. Each condition was assayed in triplicate.

Hemolysis assay

Blood (1.0 mL) was freshly collected from 4-week-old female Swiss nude mice using 1.0% heparin sodium solution

as an anticoagulant. Red blood cells (RBCs) were isolated by centrifugation at 5,000 rpm for 5 minutes. The RBC pellet was washed several times with PBS until the supernatant became colorless and then diluted in PBS to prepare a 2.0% RBC suspension (e.g., 20 μL of RBC suspension added to 0.98 mL of PBS). A 0.50 mL aliquot of the 2.0% RBC suspension was added to each of three 1.5 mL Eppendorf tubes. Next, 0.50 mL of dendrimer D or scramble RNA/D was added to the respective tubes. PBS (0.50 mL) and 1.0% Triton X-100 solution (0.50 mL) were added to separate aliquots of the 2.0% RBC suspension to serve as negative and positive controls, respectively. The samples were gently mixed and incubated at 37°C for 1 hour, followed by centrifugation at 10,000 rpm for 5 minutes. A 100 μL aliquot of the supernatant was transferred to a 96-well plate, and the absorbance at 540 nm was measured to quantify hemoglobin release. The percentage of hemolysis was calculated using the following formula:
Hemolysis (%) = [(Absorbance of sample – Absorbance of negative control) / (Absorbance of positive control – Absorbance of negative control)] × 100%.

In vitro transfection on patient-derived cells

One day prior to transfection, cells were seeded at a density of 9.0×10^4 cells per well in 6-well plates for WB or qPCR analysis, and 5,000 cells per well in 96-well plates for MTT assays. The next day, the culture medium was aspirated, and the cells were washed once with 1× PBS. For transfection, 1.0 mL of the RNA/dendrimer complex solution, prepared as described in the above section of RNA/dendrimer complex formation, was added to each well of a six-well plate, and cells were incubated at 37°C for 8 hours. After incubation, the transfection medium was replaced with complete culture medium containing 10% FBS, and the cells were maintained under standard conditions for 48 h, 72 h, or 5 days and then subjected to qPCR, WB, or MTT assays. For *in vitro* cell treatments, siRNA or saRNA was used at a final concentration of 25 nM for single-agent treatments. For combination treatments, siRNA and saRNA were each used at 25 nM. The combination index (*CI*) was calculated using the Highest Single Agent (HSA) model: $CI < 1$ was considered synergistic effect, $CI = 1$ was considered additive effect, and $CI > 1$ was considered antagonistic effect.

Synergy assessment of combinations with varying concentrations in matrix in PDCs

The synergistic effects of saRNA/D and siRNA/D combinations with varying concentrations in matrix were further evaluated on the 6 selected PDAC PDCs. PDCs were seeded in 96-well plates at a density of 4,000 cells per well and cultured for 24 h. Cells were then treated with saRNA/D and siRNA/D combinations in a 7 × 7 concentration matrix, with concentrations of 0, 5, 10, 15, 25, 35, and 45 nM for both saRNA and siRNA. RNA/D complexes were prepared as described above and added to the cells for an incubation of 8h. Subsequently, the medium was aspirated, replaced with complete medium containing 10% FBS, and the cells were cultured for an additional 5 days. Cell viability was quantified using the PrestoBlue™ reagent, with untreated cells serving as the control. Synergy scores of the combinations were calculated based on inhibition (%) normalized to the control using the HSA model implemented in SynergyFinder (version 3.0).[5] According to established criteria, synergy scores greater than 10 were considered synergistic, scores between −10 and 10 were considered additive, and scores below −10 were considered antagonistic.[6]

In vitro transfection on patient-derived organoids

PDAC cells were seeded as 8×10^4 cells per well into 6-well plate coated with 50 μl Growth Factor Reduced (GFR) Basement Membrane Matrix (Corning® Matrigel®) overnight. Then, they were treated with 1.0 mL of the RNA/D complexes, which were prepared as described in the above section of RNA/dendrimer complex formation. After 8 hours of transfection, the transfection mixture was replaced with POFM and maintained under normal growth conditions for further incubation of 72 h for WB assay and 7 days for organoids viability assay. For organoid

treatments, siRNA or saRNA were used at a final concentration of 50 nM for single-agent treatments and at 50 nM each for combination treatments. The combination index (*CI*) was calculated using the HSA and Bliss Independence (Bliss) models: $CI < 1$ was considered synergistic effect, $CI = 1$ was considered additive effect, and $CI > 1$ was considered antagonistic effect.

MTT assay for antiproliferation of RNA/dendrimer complexes

Cells were seeded at a density of 4.0×10^3 cells per well in 96-well plates. After 24h incubation in a 37°C humidified incubator, the RNA/D complexes were prepared as described above and added to the cells for an 8-hour incubation. Subsequently, the medium was aspirated, replaced with complete medium containing 10% FBS, and the cells were cultured for an additional 5 days. The MTT assay was then performed as described above.

CellTiter-Glo® 3D Cell Viability assay

1.0×10^3 cells per well were seeded into 96-well round-bottom plates, which were purchased through UGAP (Union des Groupements d'Achats Publics), a French public procurement organization, and incubated for 48 hours. The POFM was then replaced with 0.10 mL of RNA/dendrimer complexes per well, followed by incubation at 37 °C for 8 h. After transfection, the medium was exchanged with fresh POFM, and organoids were further cultured for 7 days at 37 °C in a humidified atmosphere containing 5% CO_2. Organoid viability was assessed using the CellTiter-Glo® 3D Cell Viability Assay (Promega) following the manufacturer's protocol.

Assessment of organoid morphology

Organoids were seeded at a density of 1,000 cells per well into 96-well round-bottom plates and incubated for 2 days to allow organoids formation. Subsequently, the organoids were treated with RNA/D complexes for 8 hours, followed by replacement with POFM and continued incubation for 7 days. Images of the organoids were captured using the EVOS Cell Imaging System (Invitrogen, Thermo Fisher Scientific). The diameters of the organoids were analyzed using ImageJ software.

Western blot analysis

Cells, organoids and xenograft tumors were lysed in RIPA buffer (Sigma-Aldrich, St. Louis, MO, USA) containing a protease inhibitor mixture without EDTA (Roche Applied Science, Mannheim, Germany). The samples were incubated on ice for 30 minutes to extract the proteins, followed by centrifugation at 14,000 rpm for 10 minutes at 4°C. Subsequently, protein concentration was measured by BCA Protein Assay Kit (Thermo Fischer Scientific) and proteins were separated by SDS-PAGE gradient gel, and transferred to the NC membrane after electrophoresis. NC membranes were blocked with 5% skimmed milk or 5% BSA and incubated overnight at 4°C with primary antibodies: Survivin (Cell Signaling Technology #2808), AKT2 (Cell Signaling Technology #3063), c-MYC (Abcam ab32072), p21 (Cell Signaling Technology #37543), CEBPA (Abcam ab40764), Vinculin (Abcam ab129002), Beta-actin (Abcam ab8226). The following day, after washing with 5% TBST, the membranes were incubated with secondary antibodies (Goat Anti-Rabbit IgG-HRP 4030-05, Goat Anti-Mouse IgG, Human ads-HRP 1030-05) for 45 minutes at room temperature. Signals were detected by chemiluminescence, and quantitative analysis was performed using GelPro software.

Quantitative real-time (qRT)-PCR analysis

Total RNA from cells was isolated using TRIzol reagent (Invitrogen) , and the GoScript™ reverse transcriptase kit (Promega Corporation) was used for reverse transcription. qPCR was performed on a AriaMx Real-Time PCR system. House-keeping gene 18S was used for normalization and quantification. The primers used were as follows:

AKT2-F: 5′-CAACGGGGGTGAGCTGTT-3′; AKT2-R: 5′-CGCACATCATCTCGTACATGACC-3′; MYC-F-: 5′-CCTGGTGCTCCATGAGGAGAC-3′; MYC-R: 5′-CAGACTCTGACCTTTTGCCAGG-3′; BIRC5-F: 5′-CGACGTTGCCCCCTGCCTG-3′; BIRC5-R: 5′-AAGGAAAGCGCAACCGGA-3′; CDKN1A-F: 5′-GCCCAGTGGACAGCGAGCAG-3′; CDKN1A-R: 5′-GCCGGCGTTTGGAGTGGTAGA-3′; 18S-F: 5′-CTACCACATCCAAGGAAGGC-3′; 18S-R: 5′-TTTTCGTCACTACCTCCCCG-3′; CEBPA-F: 5′-CACCGCTCCAATGCCTAC-3′; CEBPA-R: 5′-CCCATCGCAGTGAGTTCCG-3′.

Animals

All animal procedures for anticancer evaluation and in vivo imaging studies were approved by the Committee on Ethics in Animal Research (Comité d'Éthique de Marseille n°14), authorized by the Ministry of Higher Education, Research and Innovation (project authorization n°45735), and conducted in accordance with the EU Directive 2010/63/EU. Female Swiss nude mice (4 weeks old) were purchased from Charles River Laboratories, and female NOD/SCID mice (4 weeks old) were obtained from ENVIGO. All mice were housed in the Experimental Animal Facility at the Centre de Recherche en Cancérologie de Marseille, Pôle Luminy, under a 12-hour light/dark cycle. The mice were kept in enriched cages in a temperature- and humidity-controlled environment, with daily health monitoring, and were provided with *ad libitum* access to water and chow.

For the in vivo toxicity evaluation, all experimental protocols were approved by the Institutional Animal Care and Use Committee of China Pharmaceutical University, and performed in accordance with the guidelines and policies for in vivo toxicity evaluation (Approval No. 2021-06-028). Female 5-week-old ICR mice were obtained from Sino-British SIPPR/BK Lab Animal Ltd. (Shanghai, China). Throughout the study, these mice were housed at Animal Experimental Center of China Pharmaceutical University.

Patient-derived xenograft models

PDAC087T cells (1.0×10^7 cells) suspended in 50 μL PBS, or 0.05 g of PDAC079T, PDAC036T, or PDAC115T xenograft tumor mixed with 50 μL Matrigel (BD Biosciences), were subcutaneously injected into the right lower flank of each mouse under isoflurane anesthesia. Tumor growth rates differed among the models: PDAC087T required 2 weeks to reach 100 mm^3, PDAC079T and PDAC115T required 3 weeks, and PDAC036T required 4 weeks. Tumors reaching 100 mm^3 were used for antitumor efficacy evaluation.

Biodistribution of the RNA/D complex in PDX model

Nude mice bearing PDAC036T xenografts were randomly divided to three groups, and intravenously injected with Cy5-labeled scramble RNA/D, Cy5-scramble RNA alone, or PBS buffer, when tumors reached approximately 800 mm^3 within one month. At 48 hours post-administration, mice were sacrificed by cervical dislocation, and tumors and major organs such as heart, liver, spleen, kidney and lung were harvested for *ex vivo* imaging using an *in vivo* imaging system (Photon Imager; Biospace Lab). Photon counts ($ph/s/cm^2/sr$) were analyzed using M3 Version software (Biospace Lab).

Antitumor activity evaluation of combination treatments in PDX models

When tumor volumes of the PDX models of PDAC087T, PDAC079T, PDAC036T, or PDAC115T reached approximately 50-200 mm^3, mice were randomly divided into treatment groups (n = 4/5 per group) and received intravenous administration of siRNA/D or saRNA/D or siRNA/D+saRNA/D or PBS twice weekly. After eight repeated doses, all mice were euthanized by cervical dislocation. Tumor volumes were monitored throughout the treatment period and calculated using the formula: $V (mm^3) = L (mm) \times W^2 (mm^2) \times 0.5$, where L represents tumor length and W represents tumor width. The combination index (*CI*) was calculated using the HSA and Bliss

Independence (Bliss) models: *CI* below 1 was considered synergistic effect, *CI* = 1 was considered additive effect, and *CI* greater than 1 was considered antagonistic effect.

Tumors were excised and weighed. Each tumor was divided into two portions: one portion was snap-frozen in liquid nitrogen and stored at -80°C for subsequent protein extraction and WB analysis; the other portion was fixed in 4% paraformaldehyde for TUNEL assay and immunohistochemical analysis. Additionally, major organs (heart, lung, liver, kidney, and spleen) were collected and subjected to histopathological evaluation.

Inflammatory Cytokine Assay

ICR mice (6-8 weeks old, average weight 20 g) were randomly divided into four groups, with five mice per group. The mice were administered via tail vein injection with physiological saline (negative control), D, or scramble siRNA/D. The dosage of scramble siRNA was 3 mg/kg (N/P = 5). The lipopolysaccharide (LPS) group served as a positive control and received an intraperitoneal injection at a dose of 5 mg/kg. Serum samples were collected 24 hours post-administration. The concentrations of TNF-α (tumor necrosis factor-alpha), IL-6 (interleukin-6), and IL-1β (interleukin-1 beta) were measured according to the instructions of the ELISA kits (Wuhan Elabscience Biotechnology Co., Ltd., China).

Biochemical Factor Assay

Following administration as described in the inflammatory cytokine assay, serum samples were collected 24 hours post-treatment. Biochemical analyses were performed to determine the concentrations of TG, TC, BUN, CRE, TP, ALT, and AST in the serum.

Hematoxylin and eosin Staining

Organs from mice were fixed in 4.0% formaldehyde, embedded in paraffin, and sectioned into 4-μm thick slices using a Leica Histocore Biocut (Leica, Germany). The sections were then stained with hematoxylin and eosin according to the manufacturer's instructions, using a Leica Autostainer XL (Leica, Germany). Photomicrographs were captured with a ZEISS Axio Imager Z2 microscope (Zeiss, Germany) using 20× objective lens.

TUNEL Assay

Serial 4 μm sections were prepared from each paraffin-embedded tumor sample using a Leica Histocore Biocut microtome. Apoptotic cells were assessed using the TUNEL Assay Kit (ab206386), following the manufacturer's instructions. Section images were captured with a ZEISS Axio Imager Z2 microscope (Zeiss, Germany) using 20× and 40× objectives.

Immunohistochemistry

Paraffin-embedded tumor sections (4 μm thick) were incubated at 65°C for 30 minutes and then rehydrated through graded ethanol solutions. For antigen retrieval, sections were incubated in citrate buffer (TRS, pH 6; Dako) at 96°C for 20 minutes, followed by cooling at room temperature for 30 minutes. Endogenous peroxidase activity was blocked with 3% hydrogen peroxide (H_2O_2) for 10 minutes, and the sections were rinsed three times with PBS. The sections were then incubated at room temperature for 60 minutes with monoclonal rabbit anti-human Ki-67 antibody (Abcam) and monoclonal rabbit anti-human Caspase-3 antibody (Cell Signaling Technology, #9661). After three PBS washes, the sections were incubated with biotinylated goat anti-rabbit secondary antibody (Abcam) at room temperature for 30 minutes. Following additional PBS washes, Streptavidin-HRP(Agilent) was applied and incubated for 30 minutes at room temperature. Then the staining was visualized by adding diaminobenzidine from DAKO (now Agilent) for 10 min at room temperature. The slides were rinsed with distilled water, counterstained

with Mayer's hematoxylin for 30 seconds, and blued in 0.1% sodium bicarbonate solution for 3 minutes. Finally, the slides were dehydrated, cleared, and mounted with coverslips using a permanent mounting medium. Images were captured using a Zeiss Axio Imager Z2 microscope (Zeiss, Germany) 20×cobjective lens.

Combination index analysis based on HSA and Bliss models

To quantitatively evaluate the synergistic effects of the saRNA/D and siRNA/D combinations, the Highest Single Agent (HSA) and Bliss independence models were applied to saRNA/D and siRNA/D combinations tested at fixed concentrations. The treatment effect was expressed as *E*, defined as the inhibition relative to the corresponding untreatment control in this study. For *in vitro* cell and organoid studies, *E* represents the inhibition on cell proliferation or organoid viability. For *in vivo* studies, *E* represents the inhibition on tumor volume relative to the untreated control group.

For the HSA model, the combination index (*CI*) was calculated as below using the greater effect of the two single-agent treatments vs the effect obtained by the combination treatment:

$$CI_{HSA} = \frac{\max\left(E_{\text{saRNA/D}}, E_{\text{siRNA/D}}\right)}{E_{(\text{saRNA/D+siRNA/D})}}$$

For the Bliss independence model, the combination index (*CI*) was calculated as below:

$$CI_{Bliss} = \frac{E_{\text{siRNA/D}} + E_{\text{saRNA/D}} - E_{\text{siRNA/D}} \times E_{\text{saRNA/D}}}{E_{(\text{siRNA/D+saRNA/D})}}$$

where $E_{\text{saRNA/D}}$ and $E_{\text{siRNA/D}}$ represent the effects of saRNA and siRNA, respectively, delivered by dendrimer D, and $E_{(\text{saRNA/D+siRNA/D})}$ represents the observed effect of the combination treatment.

Combination effects were interpreted as follows:

- $CI < 1$: synergistic effect
- $CI = 1$: additive effect
- $CI > 1$: antagonistic effect

Statistical tests

All data are presented as the mean ± SD or mean ± SEM . Statistical analysis was conducted using one-way ANOVA, two-way ANOVA, or unpaired Student's *t*-test (GraphPad Prism 9.0.0). A p-value < 0.05 was considered statistically significant. The significance of values in the figures is indicated as follows: ns, not significant; *$P < 0.05$; **$P < 0.01$; ***$P < 0.001$; ****$P < 0.0001$.

References:

(1) Chen, J.; Ellert-Miklaszewska, A.; Garofalo, S.; Dey, A. K.; Tang, J.; Jiang, Y.; Clement, F.; Marche, P. N.; Liu, X.; Kaminska, B.; Santoni, A.; Limatola, C.; Rossi, J. J.; Zhou, J.; Peng, L. Synthesis and use of an amphiphilic dendrimer for siRNA delivery into primary immune cells. *Nat Protoc* **2021**, *16* (1), 327-351. DOI: 10.1038/s41596-020-00418-9

(2) Liu, X.; Zhou, J.; Yu, T.; Chen, C.; Cheng, Q.; Sengupta, K.; Huang, Y.; Li, H.; Liu, C.; Wang, Y.; Posocco, P.; Wang, M.; Cui, Q.; Giorgio, S.; Fermeglia, M.; Qu, F.; Pricl, S.; Shi, Y.; Liang, Z.; Rocchi, P.; Rossi, J. J.; Peng, L. Adaptive amphiphilic dendrimer-based nanoassemblies as robust and versatile siRNA delivery systems. *Angew Chem Int Ed Engl* **2014**, *53* (44), 11822-11827. DOI: 10.1002/anie.201406764

(3) Fraunhoffer, N.; Teyssedou, C.; Pessaux, P.; Bigonnet, M.; Dusetti, N.; Iovanna, J. Development of transcriptomic tools for predicting the response to individual drug of the mFOLFIRINOX regimen in patients with metastatic

pancreatic cancer. *Front Oncol* **2024**, *14*, 1437200. DOI: 10.3389/fonc.2024.1437200
(4) Schreiber, F. S.; Deramaudt, T. B.; Brunner, T. B.; Boretti, M. I.; Gooch, K. J.; Stoffers, D. A.; Bernhard, E. J.; Rustgi, A. K. Successful growth and characterization of mouse pancreatic ductal cells: functional properties of the Ki-RAS(G12V) oncogene. *Gastroenterology* **2004**, *127* (1), 250-260. DOI: 10.1053/j.gastro.2004.03.058
(5) SynergyFinder. https://tangsoftwarelab.shinyapps.io/synergyfinder/_w_56c60f6d1f094b3c8eb5910603b04cdc/#!/
(6) Li, L.; Zhang, H.; Zheng, C.; Su, Y. A review of deep learning approaches for drug synergy prediction in cancer. *npj Drug Discovery* **2025**, *2* (1). DOI: 10.1038/s44386-025-00034-1
